# Harpin Hpa1 Interacts with Aquaporin PIP1;4 to Promote the Substrate Transport and Photosynthesis in Arabidopsis

**DOI:** 10.1038/srep17207

**Published:** 2015-11-26

**Authors:** Liang Li, Hao Wang, Jorge Gago, Haiying Cui, Zhengjiang Qian, Naomi Kodama, Hongtao Ji, Shan Tian, Dan Shen, Yanjuan Chen, Fengli Sun, Zhonglan Xia, Qing Ye, Wei Sun, Jaume Flexas, Hansong Dong

**Affiliations:** 1 Department of Plant Pathology, Nanjing Agricultural University, Nanjing 210095, China; 2 Research Group on Plant Biology under Mediterranean Conditions, Departament de Biologia, Universitat de les Illes Balears, Palma de Mallorca, Illes Balears 07122, Spain; 3 Institute of Grassland Science, Northeast Normal University and National Ministry of Education Key Laboratory of Vegetation Ecology, Changchun 130024, China; 4 South China Botanical Garden, Chinese Academy of Sciences, Guangzhou 510650, China; 5 Agro-Meteorology Division, National Institute for Agro-Environmental Sciences, Tsukuba 305-8604, Japan

## Abstract

Harpin proteins produced by plant-pathogenic Gram-negative bacteria are the venerable player in regulating bacterial virulence and inducing plant growth and defenses. A major gap in these effects is plant sensing linked to cellular responses, and plant sensor for harpin Hpa1 from rice bacterial blight pathogen points to plasma membrane intrinsic protein (PIP). Here we show that Arabidopsis AtPIP1;4 is a plasma membrane sensor of Hpa1 and plays a dual role in plasma membrane permeability of CO_2_ and H_2_O. In particular, AtPIP1;4 mediates CO_2_ transport with a substantial contribute to photosynthesis and further increases this function upon interacting with Hpa1 at the plasma membrane. As a result, leaf photosynthesis rates are increased and the plant growth is enhanced in contrast to the normal process without Hpa1-AtPIP1;4 interaction. Our findings demonstrate the first case that plant sensing of a bacterial harpin protein is connected with photosynthetic physiology to regulate plant growth.

Harpins belong to a unique group of proteins secreted by the type III secretion system in plant-pathogenic Gram-negative bacteria^1^,^2^,^3^. To date, totally 23 harpins have been identified in different bacterial species and are divided into one-domain and two-domain harpins based on the unitary hydrophilic domain and an additional enzymatic domain^1^,^2^. While two-domain harpins potentially associate with the bacterial periplasm or plant cell wall (CW) to facilitate assembly of the secretion machinery, one-domain harpins target plasma membranes (PMs) to cause three distinct biological effects in a variety of plant species^4^,^5^,^6^. Hpa1 is a one-domain harpin produced by *Xanthomonas oryzae* pv. *oryzae* (*Xoo*), the pathogen that causes bacterial blight of rice *Oryza sativa* L.^2^, and performs a full repertoire of functions shared by all harpins tested so far.

One of the biological effects caused by one-domain harpins is the induction of plant immune responses. Harpins represent a special type of microbial patterns, namely invariant microbial epitopes that can be recognized by PM receptors to activate the innate immunity in plants^7^. After external application to plants or de novo expression in transgenic plants, harpins induce the apoplastic H_2_O_2_ signal and its crosstalk with intracellular pathways^8^ of signaling by phytohormones, such as salicylic acid^9^ and ethylene^10^,^11^,^12^. A linkage between apoplastic and cytoplasmic responses has been found in the ability of Hpa1 to stimulate the PM-associated NADPH oxidase and induce apoplastic H_2_O_2_, which rapidly moves into cytoplasm to regulate immunity^13^. The second effect of harpins is to induce plant growth enhancement. Following application or de novo expression, harpins enhance plant growth through cellular transduction of phytohormone signals, such as ethylene^10^ and gibberellin^14^. In Arabidopsis, Hpa1-enhanced growth associates with photosynthetic physiology and is attributable to increases of mesophyll conductance (*g*_m_) to CO_2_ and net photosynthesis (*A*_N_) rate^15^, indicating the functional linkage of Hpa1 to PIPs that may facilitate CO_2_ transport^16^,^17^. The third effect of some harpins is to serve as type III translocators, which are distinct in nature but function similarly to mediate translocation of type III effectors from bacterial cells into the cytosol of plant cells presumably by recognizing PM sensors^2^,^18^. By this mechanism, harpin-type translocators essentially contribute to bacterial virulence to host plants^1^,^2^.

Unlike the virulence role that associates with the plant-pathogen interaction process, one-domain harpins induce plant immunity and growth in a pathogen-independent manner. While the immune effect has been extensively studied, molecular mechanisms that govern the role of harpins in plant growth or bacterial virulence is less understood^1^,^2^. Pivotal questions are what plant sensors recognize harpins and how they are connected with cellular pathways. Increasing studies point Hpa1 sensors to plant PM-integral proteins. The first 60 amino acids in the 136-residue sequence of Hpa1 are critical for the three biological effects as the N-terminus-deleted version Hpa1∆NT is inactive^15^,^19^. In 22 of 23 characterized harpins^1^, N-termini contain predicted α-helical motifs that potentially determine protein-protein interactions^20^ and also direct type III translocators to eukaryotic PMs^2^. N-termini of Hpa1 and several other harpins have been shown to determine their bioactivities and recognition by plant PM-integral proteins^5^,^15^,^19^. In Arabidopsis, Hpa1 can localize to the outer surface of PM^6^ while it activates cellular signaling pathways^12^,^13^,^14^,^15^. Therefore, plant PMs must contain receptors that perceive the PM-anchored Hpa1 signal and transmit it to the cognate cellular pathways. In agreement with this hypothesis, recently we disclosed that Hpa1 expressed in yeast directly interacted with aquaporin (AQP) OsPIP1;3 from rice^2^.

AQPs are intramolecular channels essential for movements of H_2_O, CO_2_, and other small substrates across biomembranes^21^,^22^. By this role, AQPs can modulate CO_2_ uptake and assimilation (photosynthesis) in plants^23^,^24^ and regulate water relations and many other physiological processes in all living organisms^20^,^25^,^26^,^27^. In plants, AQPs fall into five major phylogenic families including the PIP family^28^. In most plant species, the PIP family comprises 13 members assigned to highly conserved PIP1 and PIP2 subfamilies, which consist of five (PIP1;1–PIP1;5) and eight (PIP2;1–PIP2;8) isoforms, respectively^28^,^29^. These proteins are believed to mediate transport of different substrates across plant PMs^29^,^30^,^31^. To date, however, only a small number of PIPs have been characterized in regard to their primary substrates and basic functions in most plants^16^,^17^,^32^,^33^. For example, Arabidopsis AtPIP1;2 facilitates CO_2_ transport in leaves^16^,^17^ and is also involved in root water relations^32^; AtPIP1;2, AtPIP2;1, and AtPIP2;6 coregulate rosette water transport^34^. These findings suggest overlapping and conserved functions of PIPs in substrate selectivity. As PMs directly face environment, PIPs are also implicated in cellular responses to a variety of extracellular signals in addition to substrate transport^2^,^21^,^29^,^32^,^35^. This functional flexibility potentially enables certain PIP isoforms to sense microbial patterns like harpins^2^,^35^.

We have explored plant sensing of Hpa1 and associated cellular pathways that regulate the bacterial virulence on rice (host plant of *Xoo*) and regulate both growth enhancement and immune responses of Arabidopsis (nonhost)^2^,^6^,^14^,^15^,^35^. This study is focused on Hpa1 sensing linked to the growth-enhancing effect in Arabidopsis. We show that AtPIP1;4 is a PM sensor of Hpa1 with a dual role in CO_2_ and H_2_O transport across the PM. It is technically infeasible to dissect proportions of AtPIP1;4-mediated transport of both substrates in contribution to Hpa1-induced plant growth enhancement. Instead, we present evidence that AtPIP1;4 increases its role in CO_2_ transport upon interacting with Hpa1, resulting in higher *A*_N_ and better growth of the plant compared to the normal process without Hpa1-AtPIP1;4 interaction.

## Results

### Hpa1 directly interacts with AtPIP1;4 at PMs of Arabidopsis cells

**Figure 1:**
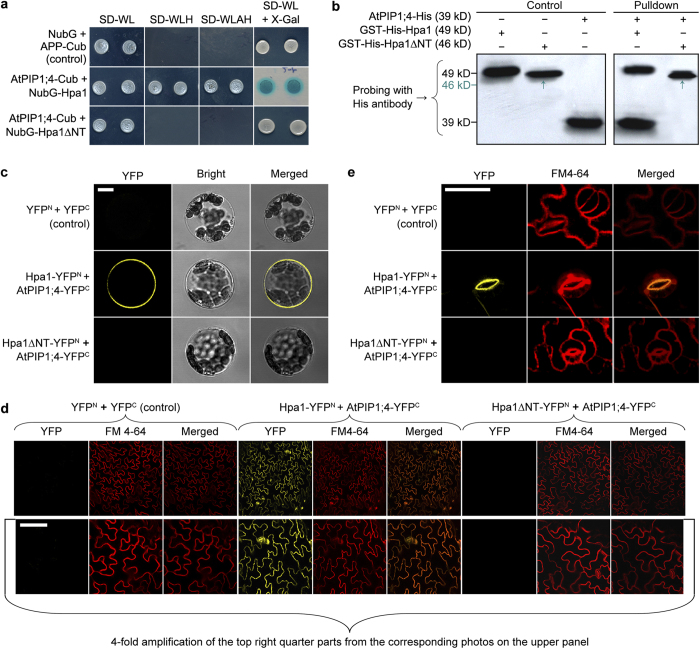
Hpa1 requires its N-terminus to interact with AtPIP1;4 in yeast, *in vitro*, and at Arabidopsis PMs. (**a**) The split-ubiquitin-based Y2H assay. Three types of synthetic dropout (SD)-amino acid nutrient media were used in screening of yeast hybrids. The SD-WL medium allows growth of yeast cells irrespectively of protein interactions. Yeast cells are able to grow on both SD-WLH and SD-WLAH media only when an interaction of tested proteins occurs. The interaction can be also detected by the X-Gal assay of colonies grown on SD-WL. (**b**) Immunoblotting of the three proteins analyzed directly (control) and proteins eluted from a glutathione-affinity resin (pulldown), showing that GST-His-Hpa1, but not GST-His-Hpa1∆NT, was able to bind with AtPIP1;4-His in the resin. (**c**–**e**) YFP BiFC imaging of (**c**) protoplasts or (**d**) and (**e**) leaves. Scale bars = 10 μm. (**c,b**) Red-fluorescent PM marker FM 4–64 was used to show cell outlines. (**e**) To better visualize BiFC signal, the guard cell was focused on the bulgy opening side.

We looked for Hpa1-interacting proteins in Arabidopsis by yeast two-hybrid (Y2H) systems. As a first step, a cDNA prey library from the Arabidopsis ecotype Col-0 was screened with the bait vector containing Hpa1 or Hpa1∆NT. Screening of yeast transformants identified seven Hpa1-interacting clones; five of them also interacted with Hpa1∆NT (Supplementary Fig. 1). The clone containing a partial sequence fragment of the *AtPIP1;4* cDNA was further studied as AtPIP1;4 was a candidate that might interact with Hpa1 at the PM. The full-length coding sequence of *AtPIP1;4* was isolated from Col-0 and retested by Y2H in crosswise combinations with Hpa1 or Hpa1∆NT as mutual bait and preys. This crosswise assay indicated AtPIP1;4 interaction with both Hpa1 and Hpa1∆NT (Supplementary Fig. 2). Proteins were further tested in a split-ubiquitin-based (SUB) Y2H system. An interaction was observed between AtPIP1;4 and Hpa1, but not between AtPIP1;4 and Hpa1∆NT (Fig. 1a; Supplementary Fig. 3). Then, Hpa1 and Hpa1∆NT were fused to histidine (His) and glutatione S-transferase (GST) tags^15^, and fusion proteins were analyzed by the *in vitro* pulldown assay. This assay detected AtPIP1;4 interaction with Hpa1 but not with Hpa1∆NT (Fig. 1b).

To locate Hpa1-AtPIP1;4 interaction in the plant cell, we carried out bimolecular fluorescence complementation (BiFC) assays with yellow-fluorescent protein (YFP). Hpa1 and Hpa1∆NT were fused to YFP N-terminal half (YFP^N^), generating the Hpa1-YFP^N^ and Hpa1∆NT-YFP^N^ fusion proteins, respectively. Meanwhile, AtPIP1;4 was fused to YFP C-terminal half (YFP^C^), forming the AtPIP1;4-YFP^C^ fusion protein. An interaction was observed between AtPIP1;4-YFP^C^ and Hpa1-YFP^N^, and the interaction was found at PMs of protoplasts (Fig. 1c; Supplementary Fig. 4) and leaf epidermal cells (Fig. 1d,e). The PM-localized Hpa1-AtPIP1;4 interaction was specific as interaction was absent in all negative controls, and it was also not present between Hpa1∆NT and AtPIP1;4 (Fig. 1c–e; Supplementary Fig. 4). Red-fluorescent PM probe FM4-64 was well colocalized with the YFP signal from Hpa1-AtPIP1;4 interaction, but colocalization was not observed in controls or between AtPIP1;4-YFP^C^ and Hpa1∆NT-YFP^N^ (Fig. 1c–e). Clearly, Hpa1 and AtPIP1;4 directly interact at the PM with the requirement for the N-terminal region of Hpa1 sequence.

### AtPIP1;4 contributes to plant growth and the promoting effect of Hpa1

**Figure 2:**
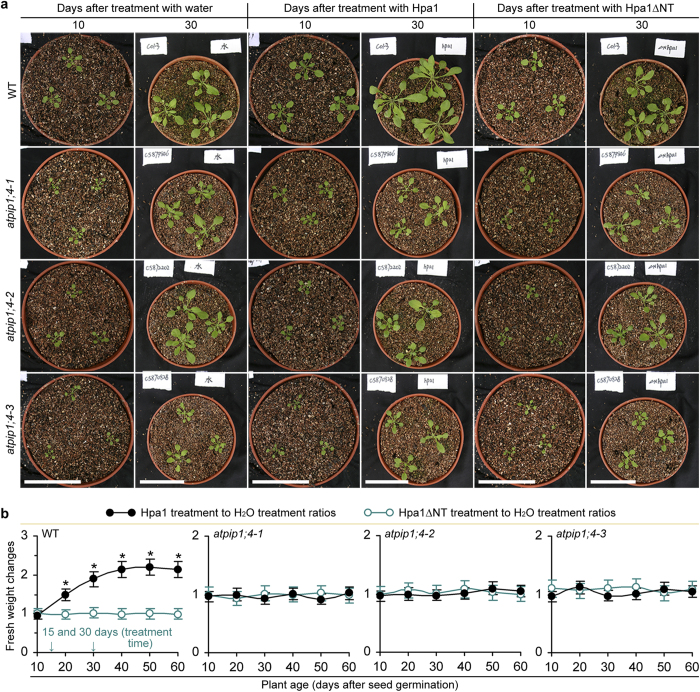
*AtPIP1;4* mutation impairs plant growth and the effect of Hpa1. (**a**) Plants photographed at the indicated times. (**b**) Plant growth comparison based on fresh weight per plant. Treatment time (tt) is indicated. Data shown are means ± SEMs (*n* = 225 plants). Asterisks indicate significant (*P* < 0.01) differences between the corresponding data pairs.

For use in studies to characterize physiological consequence of Hpa1-AtPIP1;4 interaction, we isolated homozygous lines of Arabidopsis T-DNA insertion mutants *atpip1;4-1, atpip1;4-2*, and *atpip1;4-3* (Supplementary Fig. 5a). We confirmed T-DNA-indexed coding sequence of the *AtPIP1;4* gene (Supplementary Fig. 5b) and also verified nullification of the gene expression in mutants (Supplementary Fig. 5c). By contrast, the gene was highly expressed in leaves of the wild-type (WT) plant irrespectively of treatment with water or an aqueous solution of Hpa1 or Hpa1∆NT (Supplementary Fig. 5c). This suggests that insertional mutations at the coding sequence of *AtPIP1;4* do not affect its responsiveness to Hpa1, or Hpa1 does not have a transcriptional effect on the gene. However, *AtPIP1;4* mutations caused significant (*P* < 0.01) suppressions on plant growth and the promoting role of Hpa1 (Fig. 2a,b).

Plant growth was observed in 60 days after stratification and in this period, plants were treated on 15 and 30 days with water (control) and aqueous solutions of purified Hpa1 and Hpa1∆NT, respectively (Fig. 2a). Mutants were compromised in the normal growth, and they were further impaired in Hpa1-induced growth enhancement (Fig. 2a,b). Fresh weight of WT plants was significantly (*P* < 0.01) increased in 20 days and then kept constant increase till 40 days after the first application of Hpa1 compared to Hpa1∆NT or water (Fig. 2b). The effect was not found in *atpip1;4* mutants; instead, Hpa1-treated mutant grew similarly to WT plants treated with Hpa1∆NT or water (Fig. 2a,b). Therefore, *AtPIP1;4* mutations reduce Arabidopsis growth and further arrest the promoting effect of Hpa1.

### AtPIP1;4 is required for photosynthesis and the promoting effect of Hpa1

**Figure 3:**
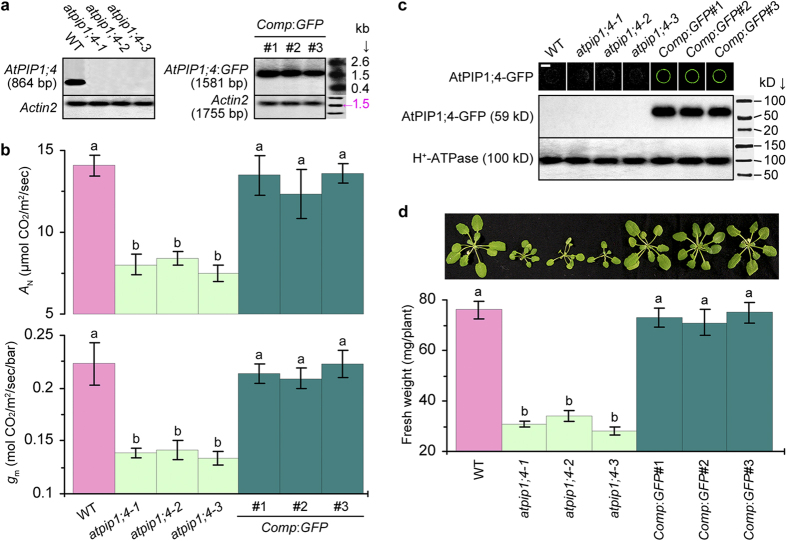
*AtPIP1;4* mutation and complementation alter CO_2_ transport and plant photosynthesis and growth. (**a**) Northern blotting analysis using *Actin2* as a reference. (**b**) *A*_N_ quantification based on gas exchange and *g*_m_ estimate based on gas exchange and chlorophyll fluorescence. Values are means ± SEMs (*n* = 18 leaves). (**c**) Protoplast imaging (scale bar = 10 μm) and immunoblotting of leaf PM fractions using H^+^-ATPase as a PM-localized protein reference. (**d**) Plant weight (means ± SEMs; *n* = 54 plants). (**b**–**d**) Different letters on error bars indicate significant (*P* < 0.01) differences.

To elucidate whether *AtPIP1;4* regulates plant growth in relation to photosynthesis, we determined *A*_N_ in WT and *atpip1;4* leaves. Nullified *AtPIP1;4* expression (Fig. 3a; Supplementary Fig. 6) caused a significant (*P* < 0.01) decrease in the *A*_N_ level (Fig. 3b; Supplementary Fig. 6). At saturating CO_2_ concentration (500 μmol/mol air) and light density (750 μmol/m^2^/s), namely photosynthetically active photon flux density (PPFD), multiples of *A*_N_ reduction in *atpip1;4-1, atpip1;4-2*, and *atpip1;4-3* vs. WT were 43%, 41%, and 46%, respectively. Thus, AtPIP1;4 occupies a >40% proportion of *A*_N_, suggesting that photosynthesis partially requires a functional *AtPIP1;4*.

As the growth (Fig. 2) and *A*_N_ (Fig. 3b) were highly reduced in *atpip1;4-3*, this mutant was used in the genetic complementation. The mutant was complemented by transformation with a recombinant vector made of full-length cDNA of the WT *AtPIP1;4* gene fused at the 5′-terminus to the gene promoter and fused at the 3′-terminus to the coding sequence of green-fluorescent protein (GFP). Complemented lines *Comp*:*GFP*#1, #2, and #3 resembled WT in *AtPIP1;4* expression (Fig. 3a; Supplementary Fig. 6). In the three *Comp*:*GFP* lines, substantial amounts of the AtPIP1;4-GFP fusion protein were found in association with PMs based on fluorescence imaging and immunoblotting analyses using PM marker protein H^+^-ATPase as a reference (Fig. 3c). These *Comp*:*GFP* lines performed similarly to resemble WT in all tested characters (Fig. 3; Supplementary Figs 6–9), indicating that the genetic complementation well restored *atpip1;4-3* to WT. In particular, *A*_N_ impaired in the mutant was retrieved by genetic complementation to approximations of WT levels (Fig. 3b), confirming the role of AtPIP1;4 in photosynthesis.

### AtPIP1;4 facilitates CO_2_ transport across PMs of plant cells

**Figure 4:**
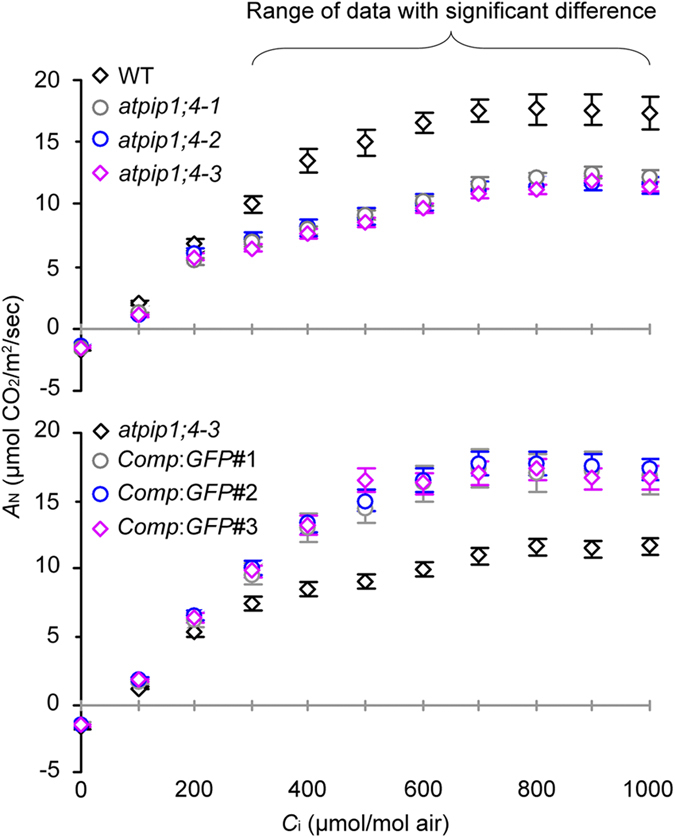
*AtPIP1;4* affects *A*_N_ response to changes in CO_2_ levels. Values are means ± SEMs (*n* = 18 leaves). Data in the range of parenthesis are significantly (*P* < 0.01) different between WT and every *atpip1;4* mutant or between *atpip1;4-3* and every *Comp:GFP* line.

As photosynthesis is limited largely by CO_2_ diffusion inside leaves and its availability at the site of photosynthesis under saturated PPFD^36^,^37^, the difference of *A*_N_ in WT, *atpip1;4*, and *Comp*:*GFP* plants presumably arose from a reduction of CO_2_ transport either by leaf stomata or by mesophyll cells or both. However, *AtPIP1;4* was unrelated to the substomatal CO_2_ concentration (*C*_i_), and to stomatal conductance (*g*_s_) either. At saturating PPFD and CO_2_ concentration, levels of *g*_s_ and *C*_i_ (Supplementary Figs 7 and 8) were similar in all plants, without evident effects introduced by *AtPIP1;4* mutation or complementation. Based on *A*_N_-*C*_i_ curve patterns (Fig. 4), *A*_N_ values were similar in all plants responding to low *C*_i_ (<200 μmol/mol air). By contrast, *A*_N_ responses to increasing *C*_i_ quantities were reduced significantly (*P* < 0.01) in mutants compared to WT or *Comp*:*GFP* plants (Fig. 4). Therefore, the role of AtPIP1;4 in *A*_N_ is likely to associate with its effect on *g*_m_, which limits transport of the intercellular CO_2_ into the cell cytosol^38^.

To test this hypothesis, we used three methods to measure *g*_m_ in *atpip1;4* mutants compared to WT or *Comp*:*GFP* plants. The *A*_N_–*C*_i_ curve-fitting method revealed that *g*_m_ values were ~41%, ~45%, and ~47% smaller accordingly in *atpip1;4-1, atpip1;4-2*, and *atpip1;4-3*, than in the WT plant (Supplementary Fig. 9). Coupled gas exchange and chlorophyll fluorescence analyses indicated that *g*_m_ values were ~41%, ~40%, and ~43% reduced in the corresponding mutants (Fig. 3b). Based on gas exchange and stable carbon isotope ^13^C discrimination, the *g*_m_ value was 45% smaller in the *atpip1;4-3* mutant than in the WT plant (Supplementary Table 1). Hence, the *g*_m_ values estimated by the three methods were consistent with each other. In essence, the *AtPIP1;4*-dependent *g*_m_ and *A*_N_ positively impacted plant growth as the growth of *atpip1;4* mutants was impaired but *Comp*:*GFP* lines grew well as did WT (Fig. 3d). These analyses suggest that AtPIP1;4 indeed is a PM facilitator for CO_2_ transport with physiological relevance to photosynthesis and growth of the plant.

**Figure 5:**
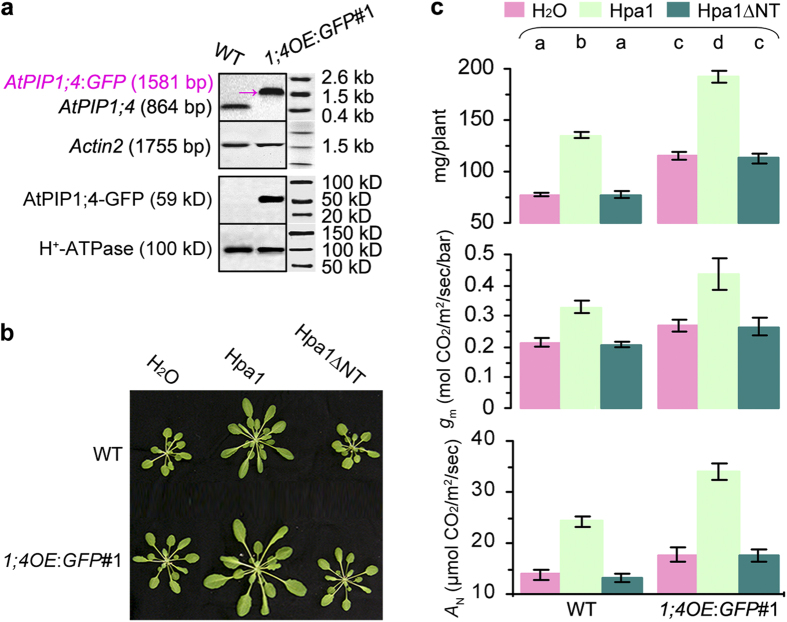
*AtPIP1;4* overexpression enhances its physiological role and the effect of Hpa1. (**a**) Northern blotting analysis using *Actin2* as a reference gene and PM protein immunoblotting with antibodies specific to the indicated proteins. (**b,c**) Fifteen-day-old plants were treated with the indicated compounds. Twenty days later, plants were photographed; fresh weight was scored (means ± SEMs; *n* = 54 plants); *g*_m_ was estimated (means ± SEMs; *n* = 18 leaves) based on gas exchange and chlorophyll fluorescence; and *A*_N_ was determined (means ± SEMs; *n* = 18 leaves) based on gas exchange. Different letters on bar graphs indicate significant (*P* < 0.01) differences.

To confirm the roles of AtPIP1;4 in *g*_m_ and *A*_N_, we determined both parameters in WT Arabidopsis plants transformed with the *AtPIP1;4*:*GFP* fusion gene and displayed *AtPIP1;4* overexpression (*1;4OE*) and *GFP* expression in the fusion form under direction by a constitutive promoter. Five *1;4OE*:*GFP* lines were screened initially based on increased growth extents in comparison with the WT plant, and *1;4OE*:*GFP*#1 acquired the best growth phenotype (Supplementary Fig. 10). Compared to the steady-state level of *AtPIP1;4* expression in the WT plant, the *AtPIP1;4*:*GFP* fusion gene was highly expressed in *1;4OE*:*GFP*#1 (Fig. 5a Northern blotting). In *1;4OE*:*GFP*#1, the AtPIP1;4-GFP fusion protein was produced at a substantial amount (Fig. 3c Western blotting). AtTTG2 overexpression resulted in significant (*P* < 0.01) enhancements in growth, *g*_m_, and *A*_N_ (Fig. 5b,c; Supplementary Fig. 10). Accordingly, the promoting effects of Hpa1 on *g*_m_, *A*_N_, and growth were increased by greater extents in *1;4OE*:*GFP*#1 than in WT (Fig. 5b,c). Therefore, the regulatory roles of AtPIP1;4 in *g*_m_ and *A*_N_ provide a physiological basis for Hpa1 to enhance Arabidopsis growth.

### AtPIP1;4 functions in H_2_O transport

**Figure 6:**
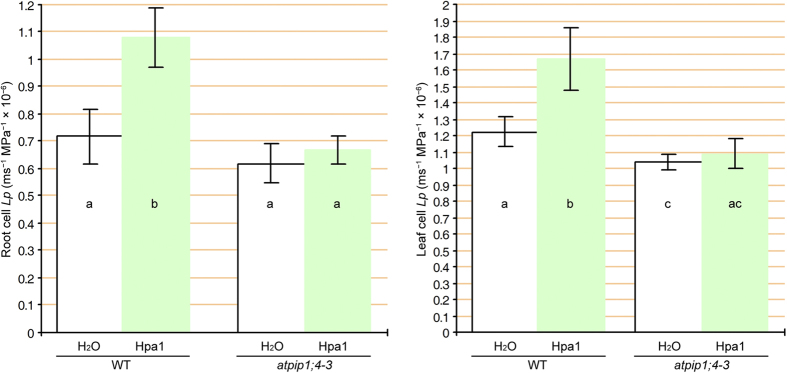
Arabidopsis cell hydraulic conductivity. Data shown are means ± SEMs (*n* = 20 cells). Different letters on error bars indicate significant (*P* < 0.05) differences.

In addition to mediating mesophyll CO_2_ conductance, AtPIP1;4 also facilitates H_2_O transport across PMs of living cells. We found that de novo expression of AtPIP1;4 was able to increase osmotic water permeability (*P*_*f*_) of African clawed frog *Xenopus laevis* oocytes. Values of *P*_*f*_ were determined to be 22.35 ± 2.85 and 17.33 ± 2.85 μm/s in oocytes following injection with cRNAs of *AtPIP1;4*:*His* and *His* used as a control, respectively. The difference in *P*_*f*_ values between *AtPIP1;4*:*His* and *His* were statistically significant (*P* < 0.01). This result was in agreement with cell pressure probe measurements^39^ performed on intact plants of Arabidopsis. In cell pressure probing assays, *atpip1;4-3* and WT plants displayed significant differences (*P* < 0.05) between each other in parameters of water relations except for cell volume and cell surface area (Supplementary Table 2). In particular, root cortical cell hydraulic conductivity (Lp_rc_) and leaf cell hydraulic conductivity (Lp_lc_) were higher in WT than in *atpip1;4-3*, with a significant difference (*P* < 0.05) in Lp_lc_ (Fig. 6). Based on the differences between WT and *atpip1;4-3* plants, AtPIP1;4 contributed to 16% (0.72 vs. 0.62) of Lp_rc_ and 37% (1.67 vs. 1.22) of Lp_lc_ (Supplementary Table 2). Evidently, AtPIP1;4 plays a role in H_2_O transport across PMs of Arabidopsis cells.

### AtPIP1;4 increases the CO_2_ transport role upon interacting with Hpa1

We sought to elucidate the physiological consequence of Hpa1-AtPIP1;4 interaction based on the primary role of AtPIP1;4 in substrate transport and the effect of Hpa1. We found that the external application of Hpa1 resulted in increases of Lp_rc_ and Lp_lc_ in the WT plant, but not in *atpip1;4-3* mutant (Fig. 6). Thus, AtPIP1;4 was responsible for the promoting role of Hpa1 on H_2_O transport. However, we felt difficult to dissect the relationship between the roles of Hpa1 or AtPIP1;4 in H_2_O transport and plant growth enhancement. At least the assumed relationship was unrelated to changes of cell size and cell surface area, which actually were similar in all plants (Supplementary Table 2). Instead, AtPIP1;4 contributes to *g*_m_ and *A*_N_ (Fig. 3) while both photosynthetic parameters are increased by the external application of Hpa1^15^. Therefore, we considered CO_2_ transport with respect to the physiological consequence of Hpa1-AtPIP1;4 interaction.

**Figure 7:**
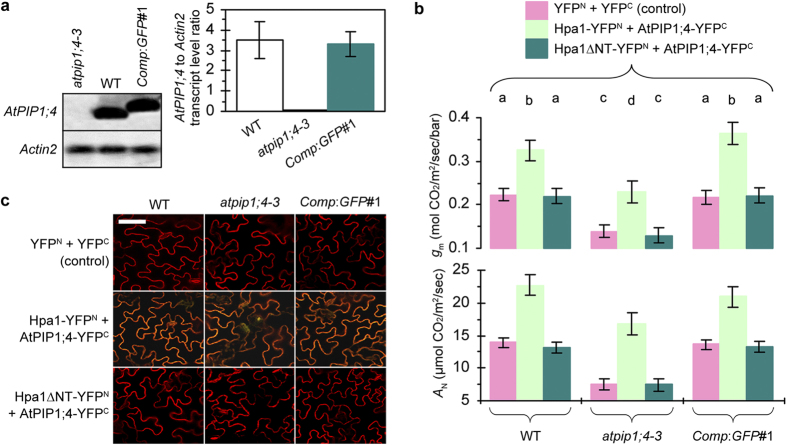
AtPIP1;4 increases its physiological role by binding de novo expressed Hpa1. (**a**) Northern blotting and real-time RT-PCR analyses of *AtPIP1;4* with the reference gene *Actin2*. The transcript ratio is shown as mean ± SEM (*n* = 6 experimental repeats). (**b**) Means ± SEMs (*n* = 18 leaves) of *g*_m_ estimate based on gas exchange and *A*_N_ determined by gas exchange and chlorophyll fluorescence at six hours after leaf transinfection with the indicated proteins. Different letters on bar graphs indicate significant (*P* < 0.01) differences. (**c**) YFP BiFC signals visualized on 60 hours after transinfection. FM 4–64 was utilized to mark cell outlines in red. Scale bar = 10 μm.

We confirmed that *AtPIP1;4* expression nullified in *atpip1;4-3* was retrieved to the WT level in *Comp*:*GFP*#1 (Fig. 7a). These plants were used for leaf transfection to elucidate whether AtPIP1;4 alters its physiological role upon binding of Hpa1. We analyzed *g*_m_ and *A*_N_ in leaves following transformation with both YFP^N^ and YFP^C^, both Hpa1∆NT-YFP^N^ and AtPIP1;4-YFP^C^, or both Hpa1-YFP^N^ and AtPIP1;4-YFP^C^. With every transformation, mutants were markedly weaker than WT or *Comp*:*GFP*#1 plants in supporting mesophyll CO_2_ conductance and leaf photosynthesis, as indicated by significantly (*P* < 0.01) smaller values of *g*_m_ and *A*_N_ in mutants (Fig. 7b; Supplementary Fig. 11). BiFC imaging showed the PM-localized interaction only between Hpa1-YFP^N^ and AtPIP1;4-YFP^C^ in all plants (Fig. 7c). In all plants, moreover, *g*_m_ and *A*_N_ were elevated significantly (*P* < 0.01) by cotransformation with AtPIP1;4-YFP^C^ and Hpa1-YFP^N^, but not with Hpa1∆NT-YFP^N^ and AtPIP1;4-YFP^C^ or YFP^N^ and YFP^C^ (Fig. 6b; Supplementary Fig. 11). Evidently, AtPIP1;4 increases its physiological role for CO_2_ transport upon interacting with Hpa1 at PMs of transfected leaves.

**Figure 8:**
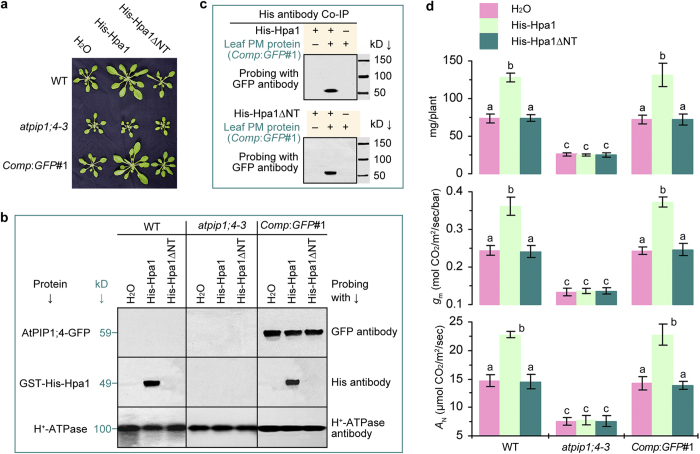
AtPIP1;4 increases its physiological role in response to externally applied Hpa1. (**a**) Plants photographed 20 days after treatment. (**b**) PM protein immunoblotting. (**c**) Co-IP analyses. **(d)** Means ± SEMs of fresh weight (*n* = 54 plants), *g*_m_ based on gas exchange plus chlorophyll fluorescence (*n* = 18 leaves), and *A*_N_ based on gas exchange (*n* = 18 leaves) in plants 20 days after treatment. Different letters on or beside error bars indicate significant (*P* < 0.01) differences.

To verify this result, we determined whether the externally applied Hpa1 was able to interact with AtPIP1;4 and affect *g*_m_ and *A*_N_. We treated 15-day-old plants by spraying over plant tops with water and aqueous solutions of His-Hpa1 and His-Hpa1∆NT, respectively (Fig. 8a), isolated leaf PM proteins, and analyzed them by immunoblotting in which H^+^-ATPase was use as a PM marker (Fig. 8b). In water or His-Hpa1∆NT treatment, none of PM proteins in the blot was able to hybridize with the antibody specific to GFP or His. In His-Hpa1 treatment, the AtPIP1;4-GFP fusion protein present in *Comp*:*GFP*#1 PM fraction was detected by hybridization with the GFP antibody. Meanwhile, probing with His antibody detected the His-Hpa1 fusion protein from leaf PM fractions of both WT and *Comp*:*GFP*#1 plants treated with His-Hpa1 in contrast to His-Hpa1∆NT or water. By contrast, both antibodies were not hybridized with PM protein samples from *atpip1;4-3* (Fig. 8b). Furthermore, co*-*immunoprecipitation (Co-IP) assays revealed AtPIP1;4-GFP interaction with His-Hpa1, but not with His-Hpa1∆NT, at *Comp*:*GFP*#1 PMs (Fig. 8c). These analyses suggest that Hpa1 existed together with AtPIP1;4 or both proteins directly interacted at PMs of WT or *Comp*:*GFP*#1 plants, but not *atpip1;4* mutant. This mutant grew similarly in different treatments but WT and *Comp*:*GFP*#1 growth was enhanced by His-Hpa1 treatment (Fig. 8a–d). In WT and *Comp*:*GFP*#1plants, His-Hpa1 treatment significantly (*P* < 0.01) elevated levels of *g*_m_ and *A*_N_, whereas, both parameters changed little in *atpip1;4-3* irrespectively of treatments (Fig. 8d). Clearly, interacting with Hpa1 enables AtPIP1;4 to boost its physiological role in CO_2_ transport and further promote photosynthesis and growth of the plant.

## Discussion

One-domain harpins are the jack of all bacterial proteins secreted by the type III secretion system, with the critical effects on bacterial virulence to host plants and both growth and immunity enhancements of nonhosts in a pathogen-independent manner^1^,^2^. With the attempt to disclose plant sensing of one-domain harpin Hpa1 and the physiological consequence, we have studied the molecular basis of Hpa1-induced Arabidopsis growth with three major results. Firstly, AtPIP1;4 is an Hpa1-interacting protein at Arabidopsis PMs (Fig. 1; Supplementary Figs S1-S4) and also a significant regulator for normal growth and Hpa1-induced growth enhancement of the plant (Fig. 2; Supplementary Fig. 10). Secondly, AtPIP1;4 plays a dual role in facilitating CO_2_ and H_2_O transport across the plant PM, occupying at least 40% of mesophyll conductance to CO_2_ (Figs 3 and 4; Supplementary Figs 9 and 11) and up to 37% of cell hydraulic conductivity in Arabidopsis leaves (Fig. 6). Thirdly, the role of AtPIP1;4 in CO_2_ transport contributes to a substantial proportion (45%) of leaf photosynthesis, and this effect is increased by AtPIP1;4 interacting with Hpa1, resulting in growth enhancement of the plant following the external application and de novo expression of Hpa1 (Figs 5, 6, 7, 8; Supplementary Fig. 11). These results discover the molecular mechanism that Hpa1 deploys to impact plant growth and photosynthetic physiology in a pathogen-independent manner.

To perform their physiological roles, AQPs must interact with their kinases for phosphorylation^34^,^40^,^41^ and may experience additional two types of hetero-molecular interactions^35^, between AQP isoforms^42^,^43^,^44^,^45^,^46^,^47^,^48^,^49^ and between AQPs and other proteins that are neither AQPs nor kinases^50^,^51^,^52^,^53^. Hetero-molecular interactions have been demonstrated for at least four of 12 characterized members of the AQP family in mammals^54^. Here, we extend this finding to plants by elucidating AtPIP1;4 interaction with Hpa1 (Fig. 1; Supplementary Figs 1-4), a one-domain harpin that relies on its N-terminal region to enhance photosynthesis and growth of Arabidopsis^15^. The N-terminus of harpins contains predicted α-helix motifs that potentially determine protein-protein interactions^20^, direct one-domain harpins to eukaryotic PMs^2^, and may serve as a determinant of Hpa1 interaction with AtPIP1;4 (Fig. 1; Supplementary Figs 3 and 4). No matter under de novo expression (Figs 1 and 6) or external application (Fig. 7), Hpa1 is able to interact with AtPIP1;4 at the PM. A frequently questioned issue is how the externally applied harpins move across plant CMs to associate with the PMs. We ever neglect plant CW architecture and proposed that one-domain harpins have the intrinsic ability to breach plant CWs^55^ and may create hole on them^2^. Then, we supposed that harpins travel through this induced hole toward the PMs and finally bind to PM sensors, followed by cellular responses^2^. In fact, this hypothesis is pointless because plant CWs are very porous and cannot block passage of large molecules, including proteins^56^. Therefore, no matter how a harpin gets access to plant surfaces, it should smoothly traverse CWs and associate with PMs or interact with a PM sensor like Hpa1 interacting with AtPIP1;4. The PM-localized Hpa1-AtPIP1;4 interaction causes a physiological consequence, i.e., increasing the primary role of AtPIP1;4 in mediating mesophyll conductance to CO_2_ and promoting leaf photosynthesis (Figs 3, 4, 5, 7, and 8; Supplementary Figs 9 and 11). The physiological role of AtPIP1;4 determines its function in normal growth and Hpa1-induced growth enhancement of the plant (Fig. 2).

PIP1;4 is one of 13 PIP isoforms identified so far in most plant species^28^,^29^,^30^,^31^ while its primary role in substrate transport is vague. Based on early studies, the expression of *AtPIP1;4* gene in Arabidopsis was induced by water deficit^25^,^57^,^58^, suggesting that the gene might function in water relations. Although PIP1-PIP2 interaction was able to increase the PIP2 permeability to H_2_O^40^,^59^, it was unclear whether PIP1s play a direct role in H_2_O transport. In rosettes of Arabidopsis grown under hydroponic conditions, *AtPIP1;2, AtPIP2;1*, and *AtPIP2;6* were highly expressed with significant contributions to water transport, whereas, *AtPIP1;4* was expressed to a lower level and might be unrelated to rosette water relations^34^. AtPIP1;4 was also tested but not definitely implicated in root H_2_O transport during lateral root growth regulated by phytohormone auxin^32^. Auxin controls lateral root growth by restricting the activity of AtPIP2;1 for mediating cell hydraulic conductivity within root tissue area at the base of lateral root primordia and beneath vascular tissues. The expression *AtPIP1;4* gene in roots is induced by lateral root growth but repressed by auxin. These data are insufficient to elucidate whether AtPIP1;4 participates in auxin-regulated growth of lateral roots. In fact, the physiological role of AtPIP1;4 is not known until now.

We elucidate the physiological role of AtPIP1;4 in association with its effect on the growth and growth-promoting effect of Hpa1 in Arabidopsis. We show that AtPIP1;4 plays a dual role in H_2_O and CO_2_ transport across PMs of the plant. Based on *A*_N_-*C*_i_ curve-fitting, gas exchange plus chlorophyll fluorescence, and ^13^C discrimination analyses, which are well accepted methods in the study of photosynthetic physiology^37^,^60^,^61^, AtPIP1;4 contributes to more than 40% of mesophyll CO_2_ conductance (Figs 3 and 4; Supplementary Figs 9 and 11). Cell pressure probe measurements suggest that AtPIP1;4 is responsible for 16% and 37% of hydraulic conductivity in roots and leaves, respectively (Fig. 6). Genetic and biochemical analyses show that AtPIP1;4 increases its CO_2_ transport role upon interacting with Hpa1 at plant PMs, resulting in increased photosynthesis rates and enhanced growth of plants compared to the normal process without Hpa1-AtPIP1;4 interaction (Figs 7 and 8; Supplementary Fig. 11). In addition, the H_2_O transport role of AtPIP1;4 may also contribute to Hpa1-induced plant growth enhancement as this role increases in plants treated with Hpa1 (Fig. 6). At present, however, we don’t have evidence to support this hypothesis.

In contrast to the widely accepted theory of H_2_O transport role as initially assigned to AQPs, PIP-facilitated CO_2_ transport has been under debate^62^,^63^,^64^,^65^ whilst the physiological role of individual AQP isoforms is also a matter of controversy^28^,^66^. Recent studies support the significance of PIP1s in CO_2_ transport^17^,^57^,^67^,^68^ and in Arabidopsis, both AtPIP1;2^17^,^67^ and AtPIP1;4 (Figs 3-5) have been characterized as physiologically relevant facilitators of CO_2_ transport across PMs. However, none of the PIP1s play a full role in mediating CO_2_ transport. Instead, mutants or gene-silenced plants are still able to assimilate CO_2_ without chlorosis. Presumably, additional PIPs or other channels also govern transport of CO_2_ across PMs, and they may function as a consortium to implement a full function in the process^24^. This claim agrees with recent findings on the functional specificity and redundancy of PIPs. For example, AtPIP1;2, AtPIP2;1, and AtPIP2;6 share their functions in leaf water relations^32^,^34^, AtPIP2;1 also regulates cell hydraulic conductivity in roots^32^, and AtPIP1;4 functions in both roots and leaves to regulate hydraulic conductivity (Fig. 6). As one more example, tobacco *Nicotiana tabacum* NtAQP1 is unrelated to the shoot H_2_O transport; instead, it increases *g*_m_ and *A*_N_, resulting in enhanced plant growth^40^. In addition, the predicted NtAQP1 protein (accession number CAA04750) does not share similarities with any Arabidopsis AQPs, except for an 88% identity in two short regions (2/3–181/182 and 183/182–287/286) to a hypothetical protein (accession number EFH49212.1). Hence, AQPs are highly diverse with overlapped and redundant functions in plants^21^,^28^, explaining the dual role of AtPIP;4 in CO_2_ and H_2_O transport.

In summary, our data offer robust evidence for the molecular mechanism by which one-domain harpin Hpa1 interacts with AtPIP1;4 to facilitate CO_2_ transport in Arabidopsis. This finding should stimulate further studies to explore the structural basis of AQP-partnering protein interactions^35^. AQPs possess six α-helical TM (TM1–TM6) domains that are tilted along the plane of PM and linked one to the other by five connecting loops (LA–LE)^21^,^29^, LB, LD, and both N-terminal and C-terminal regions locate inside the cell and potentially bind to cytosolic substrates^51^,^69^. Inversely, LA, LC, and LE face the apoplasm and have the opportunity to contact with apoplastic substrates^70^. Presumably, LA, LC, and LE enable PIPs to sense biotic signals and therefore extend their functional scopes beyond substrate transport^42^,^71^,^72^,^73^. This structural feature and functional flexibility of AQPs provide the molecular basis of AtPIP1;4 interaction with Hpa1 and the subsequent effect on photosynthesis. Studies in the future to characterize whether the topological distribution of AtPIP1;4 on the PM alters upon interacting with Hpa1 will be critical to elucidate mechanisms that underpin Hpa1-AtPIP1;4 interaction and the physiological consequence.

## Methods

### Plant material and growth conditions

Seeds of Arabidopsis ecotypes Col-0 and Col-3 (stock numbers CS28166 and CS28171) and Col-3 mutants *atpip1;4-1, atpip1;4-2*, and *atpip1;4-3* (CS879846, CS872202, and CS870828) were purchased from The Arabidopsis Information Resource (TAIR, www.arabidopsis.org). Homozygous mutants were isolated, transgenic plants generated, and all plant seeds maintained in H.D. lab. Seeds were germinated in flat plastic trays filled with a substrate containing peat, sand, and vermiculite (1:1:1 v/v). Three days later, germinal seedlings were moved into 60-ml pots (3 plants per pot) filled with the same substrate. Seeds were incubated and plants were grown in plant growth chambers under 24 ± 1 °C and 12-hour light at 250 ± 50 μmol quanta/m^2^/sec.

### Protein interaction assays

Y2H system III (Clontech) was used in screening of cDNA prey library CD4-22 from Arabidopsis ecotype Col-0 (TAIR) with a bait vector containing the *hpa1* or *hpa1∆NT* gene^15^. Positive clones were sequenced and retested in the system. The positive clone containing a partial sequence fragment of *AtPIP1;4* was further tested in pairwise combination with Hpa1 or Hpa1∆NT as mutual bait and preys. Full-length *AtPIP1;4* cDNA was obtained by RT-PCR with mRNA isolated from leaves of Arabidopsis ecotypes Col-0 and Col-3. Sequences of both RT-PCR products were confirmed to be 100% identical with each other and with the published sequences. AtPIP1;4 in combination with Hpa1 or Hpa1∆NT was tested in SUB Y2H system (Dualsystems). This system was also employed to test protein combinations between Hpa1 as bait and each of OsPIP1;1–1;3 isoforms as a prey. To carry out *in vitro* pulldown assays, AtPIP1;4 was linked to a His_(6)_ tag while Hpa1 or Hpa1∆NT was fused to both His_(6)_ and GST tags^4^,^15^. The AtPIP1;4-His fusion protein was produced in *Pichia pastoris*^74^ while GST-His-Hpa1 and GST-His-Hpa1∆NT were produced in *Escherichia coli*^4^. Proteins were purified by nickel chromatography^4^ and used in pulldown assays^74^. Co-IP was performed on leaf PM fraction by using the Pierce^®^ Co-IP Kit (Thermo Sci.) as per the manufacturer’s instruction. For *in vivo* molecular interaction analyses and other tests, 35-day-old plants and their fourth leaves were used unless specified elsewhere. YFP BiFC tests were conducted on leaves and leaf protoplasts^75^,^76^. Cell outlines were visualized by a 5 μg/ml aqueous solution of PM marker FM4-64 (Invitrogen), which was applied by immersing protoplasts or leaves in tubers on ice within two minutes before confocal microscopy. The FM4-64 signal was captured with 734-nm emission and 558-nm excitation while the YFP signal was captured as previously described^75^.

### Mutant screening

T-DNA-insertional Arabidopsis mutants *atpip1;4-1, atpip2;4-2*, and *atpip1;4-3* were generated previously by transformation of Col-3 with plant binary vector pDAP101 (TAIR). This vector carries the Basta-resistant gene (*Basta*^*r*^) as a selective marker. Basta is a commercial brand name of the herbicide N-phosphonomethyl glyline, namely glyphosate. A commercial supply of Basta as a 10% glyphosate aqueous solution (Bio Basic Inc.) was used in screening of *atpip1;4* mutants on the basis of their heterozygous T2 seeds initially provided by TAIR. A water-diluted solution of the Basta product at the final concentration of 0.5% glyphosate (v/v) was applied twice a week by the aid of an atomizer to spray over tops of 10–35-day-old mutant plants in T3 and T4 generations. Plant growth was monitored; rates of plant survival were scored; seeds produced by plant individuals were harvested separately; and all seeds from a single plant were coded as a single seed stock. For every mutant, homozygous progenies were identified by the criterion that all of T4 plants, at least 10 individuals, derived from a single stock of T3 seeds were resistant to Basta, growing well and producing viable seeds. Those T4 plants were regarded to be homozygous at the *Basta*^*r*^ locus and hypothetically at the locus of T-DNA insertion as well. Homozygosis at both T-DNA insert and *Basta*^*r*^ loci was confirmed by PCR analyses with specific primers and the genomic DNA isolated separately from 10 individuals of T4 plants grown from the same stock of T3 seeds. PCR products were confirmed by sequencing and alignment comparisons through the NCBI Blast tool (http://blast.ncbi.nlm.nih.gov/Blast.cgi). Sequencing information was also used to confirm correct orientation of the *Basta*^*r*^ gene and *AtPIP1;4*-flanking T-DNA sequence in the vector integrated into the plant genome. If all of 10 plant individuals in T4 generation derived from a single stock of T3 seeds were resistant to Basta and contained the T-DNA insert and *Basta*^*r*^ gene at correct sites, seeds in the stock were regarded as homozygous at the mutation locus and their progenies were used subsequently in all of the experiments.

### Genetic complementation

The genetic complementation unit was constructed with the plant binary pCAMBIA1301 vector (CAMBIA), which contains a *GFP* gene and the cauliflower mosaic virus the cauliflower mosaic virus 35S promoter (*P35S*). The promoter region and coding sequence of *AtPIP1;4* were cloned from Col-3 DNA and RNA by PCR and RT-PCR, respectively. Their sequences were confirmed by sequencing with clones in the pMD19-T vector (Takara). Confirmed *AtPIP1;4* promoter and cDNA sequences were inserted into pCAMBIA1301 at the front of *P35S* and the right border to form the genetic complementation union and exclude *P35S* in the recombinant vector. The recombinant vector was transferred into cells of the *Agrobacterium tumefaciens* strain EHA105, and a suspension of recombinant EHA105 cells was used in transformation of *atpip1;4-3* through blossom infiltration^77^. Transgenic plants were screened and characterized as previously described^77^,^78^ and T3 homozygous progenies were used in this study.

### Gene overexpression

The *AtPIP1;4:GFP* fusion gene was inserted into the plant binary vector pCAMBIA1301 between the 3′-terminal end of *P35S* and front of the right border^77^. The recombinant vector was transferred into the genome of WT Col-3 plants under mediation by *A. tumefaciens*^13^. Transgenic plants were created, screened, and characterized by conventional protocols^12^,^77^. T3 homozygous progenies were used in this study. Gene overexpression was verified by real-time RT-PCR and Northern blotting analyses, and production of the AtPIP1;4-GFP fusion protein was detected by immunoblotting with specific GFP antibody (Novagen) or His antibody (Merk). In immunoblotting analyses, PM marker protein H^+^-ATPase was as a reference and probed with the specific antibody (Santa Cruz).

### Plant treatment and growth scoring

Prokaryotic expression vectors used for production of Hpa1-His and Hpa1∆NT-His fusion proteins were constructed previously^15^. Proteins were produced in *E. coli*, purified by nickel chromatography, and treated with an enterokinase to remove His^4^. Purified proteins were prepared as aqueous solution stocks and their concentrations were determined^4^. Based on known effective dosage of harpins^4^,^15^,^16^,^78^, every protein was used at a final concentration of 10 μg/ml in an a water-diluted solution and applied by the aid of an atomizer to spray over tops of plants on 15 and 30 days after stratification. Plants were treated similarly with pure water in control. Plant growth extents were quantified as fresh weight at time intervals after treatment.

### Gene expression analysis

Information on genes tested in this study is provided in Supplementary Table 3. Previously described methods^4^ were used in Northern blotting, RT-PCR, and real-time RT-PCR analyses for *AtPIP1;4* expression. The constitutively expressed *EF1α* and *Actin2* genes were used as references. In real-time RT-PCR, cDNA templates were analyzed together with temple-absent controls. Relative level of *AtPIP1,4* expression was quantified as the transcript quantity ratio of *AtPIP1;4* to a reference gene.

### Immunoblotting

Leaf and cytoplasmic PM proteins were isolated^13^ and analyzed by immunoblotting^79^. Protein blots were incubated with every of the specific antibodies and hybridized to horseradish peroxidase-conjugated goat antimouse immunoglobulin G from the BeyoECL Plus kit (Beyotime).

### Gas exchange measurements

Plants used in gas exchange measurements were grown in substrate-overfilled pots^61^ for 35 days and measurements were performed on top third and fourth fully unfolded leaves unless specified elsewhere. Leaf gas exchange was measured with the Li-6400XT portable photosynthesis system and the equipped 2-cm^2^ leaf chamber (Li-Cor. Biosci.). Detailed measurements on single leaves were performed by following the manufacturer’s instructions and previously described experimental procedures^61^. During measurements, relative humidity in leaf chamber was constantly maintained at 45% and leaf temperature was kept at 25 °C. CO_2_ concentrations at the inlet and outlet of the leaf chamber were monitored by the non-dispersive infrared gas analyzer installed in the system. PPFD was controlled by adjusting intensities of the lamp-house irradiation. Readings of *A*_N_, *C*_i_, and more related photosynthetic parameters were documented automatically by the S-501 digital monitor integrated into the Li-6400XT system. For every plant genotype or treatment, instantaneous gas exchange measurements on a single leaf were performed every two hours in the light cycle of plant growth to obtain sufficient and reliable data, which were used subsequently in estimation of *g*_m_ by curve-fitting, chlorophyll fluorescence quenching, and ^13^C discrimination analyses^80^,^81^.

### *g*_m_ estimate by the *A*_N_-*C*_i_ curve-fitting method

Assessments of *g*_m_ by the curve-fitting method require a large number of data points to be reliable^82^,^83^, it was only possible to perform this estimation in young leaves^37^,^61^. Therefore, photosynthesis responses to changes in light intensity and CO_2_ concentration were analyzed through gas exchange measurements on top 3–5 expanded leaves of 35-day-old plants. *A*_N_–*C*_i_ curves were established by measuring *A*_N_ at PPFD 750 μmol/m^2^/sec and a range of *C*_i_ (1000–0 μmol/mol air on a 100-descendent gradient). The value of *g*_m_ was estimated by the *A*_N_–*C*_i_ curve-fitting method^82^.

### *g*_m_ estimate based on gas exchange and chlorophyll fluorescence

Gas exchange measurements coupled with chloroplast fluorescence monitoring were performed on top 3–5 expanded leaves of 35-day-old plants. Gas exchange was measured under saturating light (PPFD 750 μmol/m^2^/sec) and CO_2_ concentration of 500 μmol/mol air. The respiration rate in light (*R*_L_) and CO_2_ photocompensation point in the absence of R_L_ (Γ*) were estimated by a previously described method^37^. Then, both parameters were used in *g*_m_ assessments together with the photosynthetic electron transport rate (*J*) obtained from the chlorophyll fluorescence quenching analysis^61^.

Chlorophyll fluorescence was analyzed with the Multiple Excitation Wavelength Chlorophyll Fluorescence Analyzer (Heinz Walz GmbH). This analyzer was operated under conditions of the leaf temperature kept at 25 °C, the chamber CO_2_ concentration adjusted to 500 μmol/mol air, and the leaf-to-air vapor pressure deficit maintained at 1.2 kPa. Leaves were first adapted to dark for 20 minutes and then adjusted to PPFD 1500 μmol/m^2^/sec, and chlorophyll fluorescence was measured on three 3-mm^2^ circular bars located between leaf nervures in a single leaf. The value of *J* from the fluorescence analyzer (*J*_flu_) and the photosynthetic parameters *A*_N_, R_L_, and Γ* obtained from gas exchange measurements were utilized to estimate *g*_m_ using equation (1)^61^:


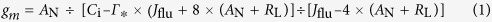


### *g*_m_ Estimate by ^13^C Discrimination in Recently Synthesized Carbohydrates

Because *g*_m_ assessments based on gas exchange in leaves and stable carbon isotope discrimination in recently synthesized carbohydrates^37^ require a large amount of pulverized and lyophilized leaf material (100 mg per sample)^84^,^85^, the experiments were conducted with large-scaled pools of plant populations^86^. For every Arabidopsis genotype, therefore, 300 plants in 100 pots were grown for 35 days under the same conditions. Totally 210 plants of a genotype were chosen based on the criterion of uniform growth and used as a material pool for gas exchange and ^13^C discrimination analyses. Immediately after gas exchange measurements performed on top 3–5 expanded leaves of 10 plant individuals randomly selected from the material pool, equivalent leaves were excised from all of the 210 plants and pulverized thoroughly with liquid nitrogen. Resulting leaf powders were lyophilized, weighed, stored at –65 °C when necessary, and processed (directly or after thaw) to prepare soluble sugars, which were subsequently used for ^13^C discrimination (∆) in recently synthesized carbohydrates as previously described^84^,^85^.

Soluble sugars were extracted from low-molecular weight compounds (LMWC), which was isolated from lyophilized leaf powers and purified by ion-exchange chromatography. A critical step in LMWC purification was ion exchange chromatography with the cation-exchange resin DOWEX 50W and the anion-exchange resin DOWEX 1. The former resin was used for the separation of amino acids from organic acids and sugars, and the latter was used to separate organic acids from soluble sugars^87^. Concentrations of ^13^C in purified sugar preparations and in source air as well were determined by coupled analysis with an element analyzer (EA) and an isotope ratio mass spectrometer (IRMS)^88^. The IRMS facility used in this analysis is a Finnigan MAT Deltaplus XP isotope ratio mass spectrometer (Thermo Finnigan MAT), which was coupled to a Flash EA 1112 Series elemental analyzer (Thermo Italy) through a six-port valve and a universal interface for EA-IRMS coupling (ConFlo III, Thermo Finnigan). With readings from the Flash EA analyzer, the observed Δ (Δ_Obs_) was calculated using equation (2)^84^:


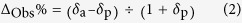


Here, δ_a_ and δ_p_ are the isotope compositions of source air and plant material, respectively, relative to international standard Vienna-Pee Dee Belemnite^87^.

Values of *g*_m_ were determined by comparing Δ_Obs_ with predicted discrimination (Δ_i_)^60^. Δ_i_ was calculated with empirical and observed parameters^88^ as follows: *a* (empirical proxy 4.4‰) was the fractionation during diffusion in air; *b* (28.2%) was the discrimination associated to carboxylation reactions; *C*_i_ was obtained from the gas exchange measurement; and *C*_a_ was the air CO_2_ concentration adjusted during the measurement. Then, Δ_i_ was calculated using equation (3)^37^:


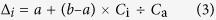


In addition to Δ, discrimination parameters also required for estimation of *g*_m_ were empirical values^89^ of the fractionation during the dissolution of CO_2_ (*e*_s_; 1.1%) and the discrimination by CO_2_ diffusion in the liquid phase (*a*_1_; proxy 0.7%) and by photorespiration (*f*; 8%). These discrimination parameters were used along with gas exchange parameters Γ*, the dark respiration rate (*R*_D_), and the carboxylation efficiency (*k*) to calculate *g*_m_ according to equation (4)^37^:


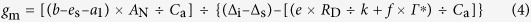


Values of *g*_m_ from this calculation and estimated by the *A*_N_-*C*_i_ curve-fitting method were compared to evaluate the reliability of *g*_m_ assessments based on the gas exchange and isotope discrimination protocol.

### Oocyte *P*_*f*_ determination

For expression in *X. laevis* oocytes, capped cRNA of *PIP1;4:His* or *His* was synthesized *in vitro* from *Not* I-linearized pGH19 plasmid and purified with the RNeasy Mini kit (Qiagen). Stage IV–V oocytes were defolliculated and injected with 5 ng of cRNA or 50 nl of diethyl pyrocarbonate-treated water in control. Injected oocytes were incubated for 2–3 days at 18 °C in ND96 culture medium^89^. *P*_f_ was estimated by the oocyte swelling assay^90^. Oocytes were transferred into liquid ND96 medium diluted to 50 milliosmolar with distilled water, and the time course of volume increase was monitored at room temperature by videomicroscopy with an on-line computer^91^.

### Measurements of cell hydraulic parameters

Lp_rc_ and Lp_lc_ were determined by cell pressure probe (CPP) measurements performed on root segments and leaf blades^31^. Pulled glass microcapillaries were beveled to a tip diameter of 5–7 μm, filled with type AS4 silicon oil (Wacker), and mounted vertically on a pressure probe. For Lp_rc_ measurement, root segment was excised from plants grown in hydroponic conditions, and was placed on a metal sledge that was covered with filter paper. An aerated plant culturing solution was circulated along the root segment to maintain hydration. Cortical cells from second to fourth layer and at 5–8 cm distance from the root apex were punctured using a CPP. Cell turgor was restored by gently pushing the meniscus to a position close to the surface of the root, and the values of cell turgor pressure were recorded by a computer. The half time (T_1/2_) of hydrostatic water flow across cell membrane, which is inversely proportional to cell hydraulic conductivity (T_1/2_ ∞ 1/Lp) was obtained from pressure relaxation curves with the aid of the probe. T_1/2_ measurements for a given cell were finished within 10 min after root excision. Lp_rc_ was calculated from the measured T_1/2_. For Lp_lc_ measurement, a mature young leaf blade on the plant was fixed onto a metal support and leaf cells were punctured using a CPP. Upon a successful puncture, cell turgor was restored by gently moving the meniscus to a position close to the surface of the leaf. Lp_lc_ was determined as for roots^31^.

### Statistical analysis

All experiments were repeated at least three times with similar results. Quantitative data were analyzed with IBM SPSS19.0 software package^92^. Homogeneity-of-variance in data was determined by Levene test, and formal distribution pattern of the data was confirmed by Kolmogorov-Smirnov test and P-P Plots. Data were subjected to analysis of variance along with Fisher’s least significant difference test.

## Additional Information

**How to cite this article**: Li, L. *et al.* Harpin Hpa1 Interacts with Aquaporin PIP1;4 to Promote the Substrate Transport and Photosynthesis in Arabidopsis. *Sci. Rep.* **5**, 17207; doi: 10.1038/srep17207 (2015).

## Supplementary Material

Supplementary Information
