# Supplementary material for: Harpin Hpa1 Interacts with Aquaporin PIP1;4 to Promote the Substrate Transport and Photosynthesis in Arabidopsis
Source: Sci Rep. 2015 Nov 26;5:17207. doi: 10.1038/srep17207 (PMC4660436; doi:10.1038/srep17207)
Supplement: Supplementary Information [file srep17207-s1.doc]

**Harpin Hpa1 Interacts with Aquaporin PIP1;4 to Promote the Substrate Transport and Photosynthesis in Arabidopsis**

Liang Li, Hao Wang, Jorge Gago, Haiying Cui, Zhengjiang Qian, Naomi Kodama, Hongtao Ji, Shan Tian, Dan Shen, Yanjuan Chen, Fengli Sun, Zhonglan Xia, Qing Ye, Wei Sun, Jaume Flexas & Hansong Dong

**Supplementary Information**

**
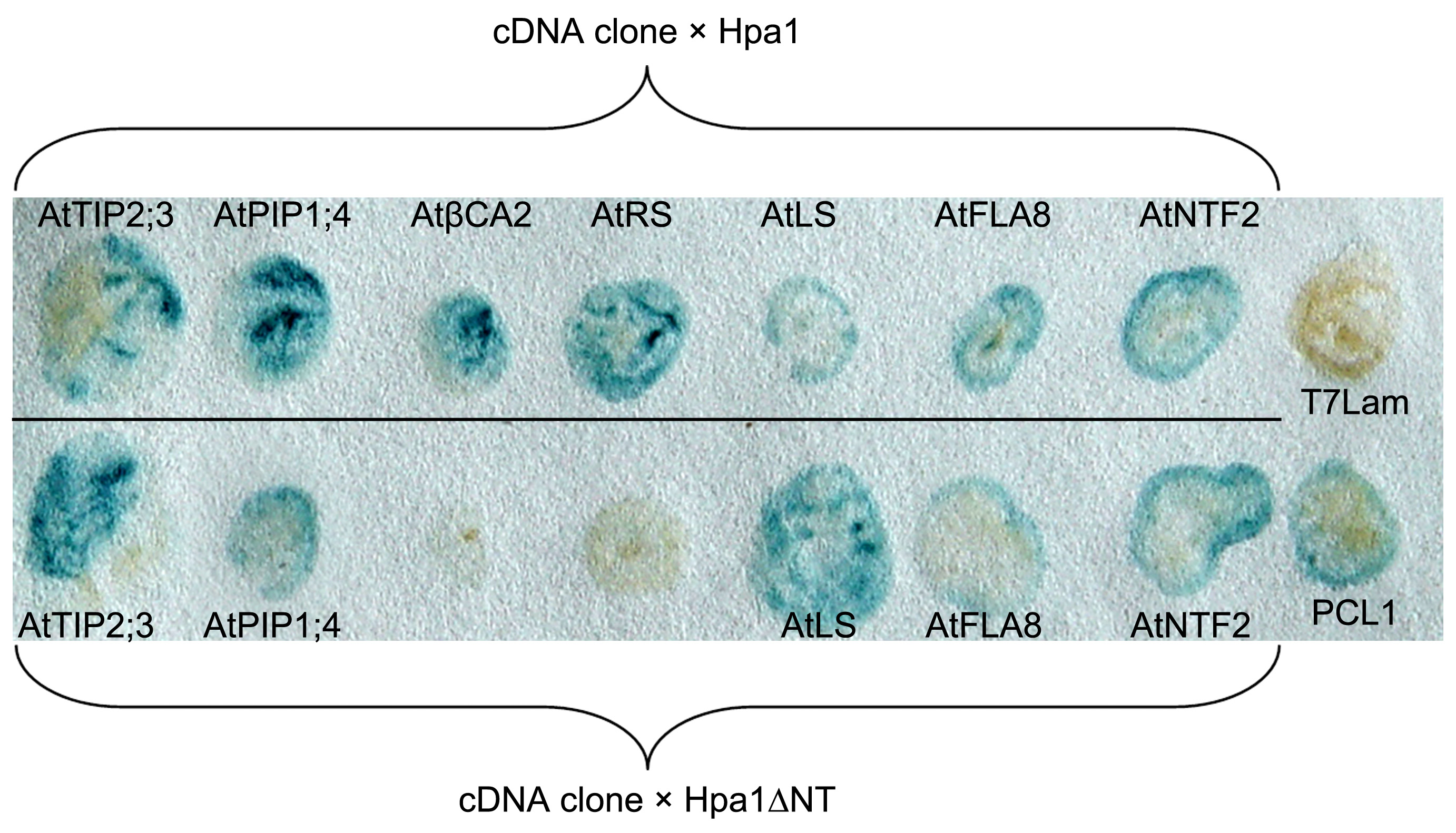
**

**Supplementary Figure 1 | Identification of Hpa1-interacting proteins in Arabidopsis.** A prey cDNA library from the Arabidopsis ecotype Col-0 was screened with full-length Hpa1 or the N-terminus-deleted version Hpa1∆NT by the conventional (not split-ubiquitin-based) yeast two-hybrid (Y2H) system. Yeast colonies represent positive cDNA clones that interact with Hpa1 and/or Hpa1∆NT. PCL1 and T7Lam are positive and negative controls, respectively. In the confirmative test, positive clones were isolated from yeast, retransformed into yeast cells harboring the *hpa1* or *hpa1∆NT* gene cloned in a bait vector, and retested in the Y2H system. Confirmed positive clones contain AtTIP2;3 (tonoplast intrinsic protein; GenBank accession number AED95514; AT4G00430), AtPIP1;4 (plasma membrane intrinsic protein), AtβCA2 (carbonic anhydrase; AED92068), AtRS (riboflavin synthase; AEC07057), AtLS (lumazine synthase; O80575), AtFLA8 (fasciclin-like arabinogalactan-protein 8; AEC10559), and AtNTF2 (nuclear transport factor 2; AC027656_16). AtTIP2;3 and AtPIP1;4 locate at the tonoplast membrane and plasma membrane (PM), respectively, and the other five proteins are all cytoplasmic. AtPIP1;4 is most possible to interact with Hpa1 at the PM, whereas, TIP2;3 is not likely to interact with Hpa1 in planta because Hpa1 localizes to the PM and does not have the opportunity to contact with TIP2;3 under our experimental conditions. Therefore, AtPIP1;4 but not TIP2;3 was further studied.

**
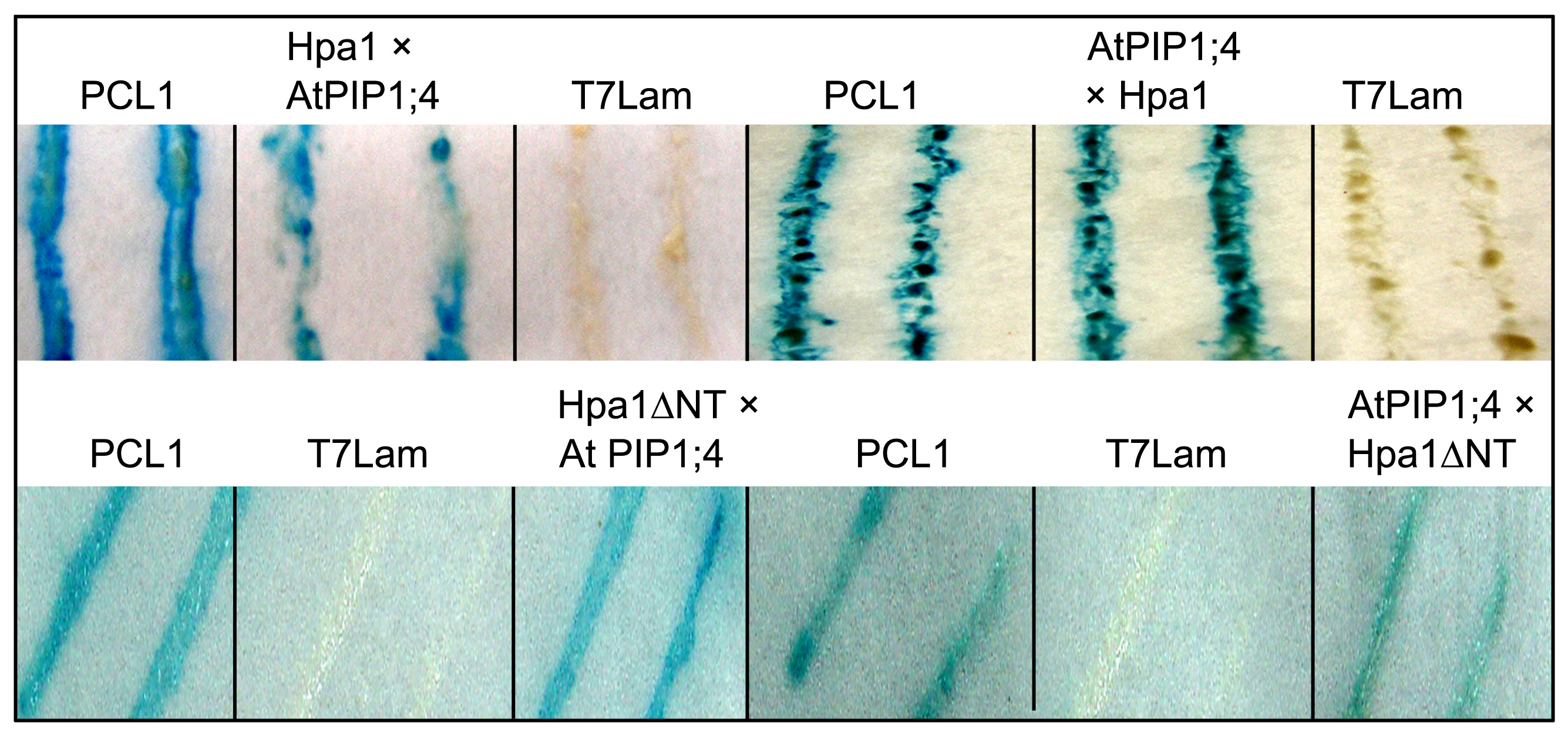
**

**Supplementary Figure 2** **| Crosswise Y2H tests of AtPIP1;4 in combinations with Hpa1 and Hpa1∆NT.** The full-length coding sequence of *AtPIP1;4* from Col-0 was tested by the conventional Y2H system in crosswise combinations with Hpa1 or Hpa1∆NT as mutual bait and preys.


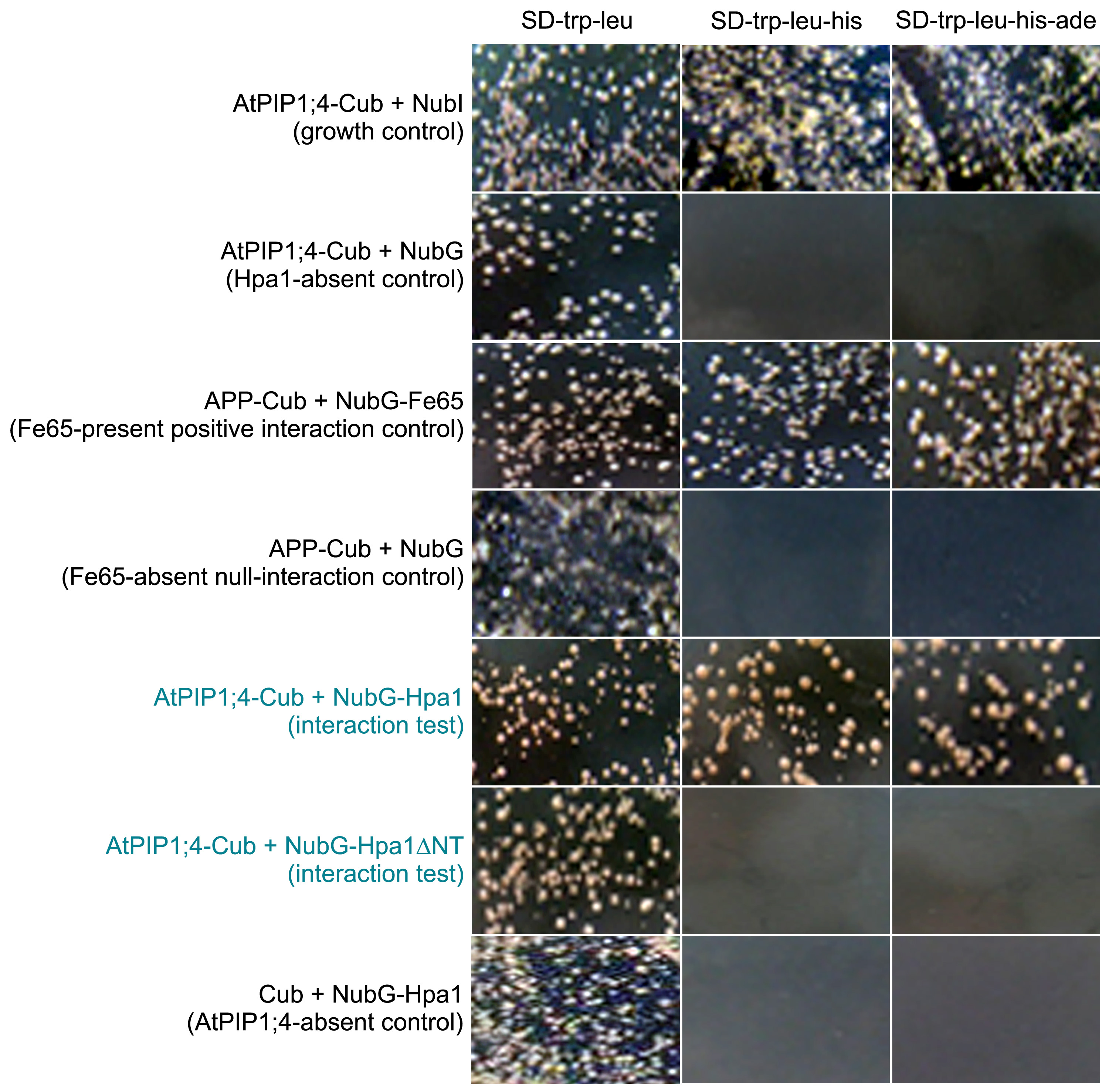


**Supplementary Figure 3 | SUB Y2H assays for AtPIP1;4-Hpal interaction in comparison with multiple controls.** As shown on left, Cub refers to the C-terminal half of ubiquitin; NubI denotes the N-terminal half of ubiquitin made as the wild-type (WT) version carrying an isoleucine at position 3; NubG is a NubI mutant due to isoleucine substitution with glycine; Fe65 is a cytosolic protein; APP refers to the amyloid A4 precursor protein. The AtPIP1;4-Cub fusion protein was tested with NubI in a yeast growth control. Combinations of APP-Cub with NubG and NubG-Fe65 were used as negative and positive interaction controls, respectively. In addition, AtPIP1;4-absent and Hpa1-absent controls were also included. As shown on top, three types of synthetic dropout (SD)-amino acid nutrient media were used in screening of yeast hybrids. Yeast growth was evaluated by incubation on the SD-trp-leu medium. This medium allows growth of yeast cells with and without protein interactions. By contrast, only yeast cells in which tested proteins had interacted are able to grown on both the SD-trp-leu-his and SD-trp-leu-his-ade media.

**
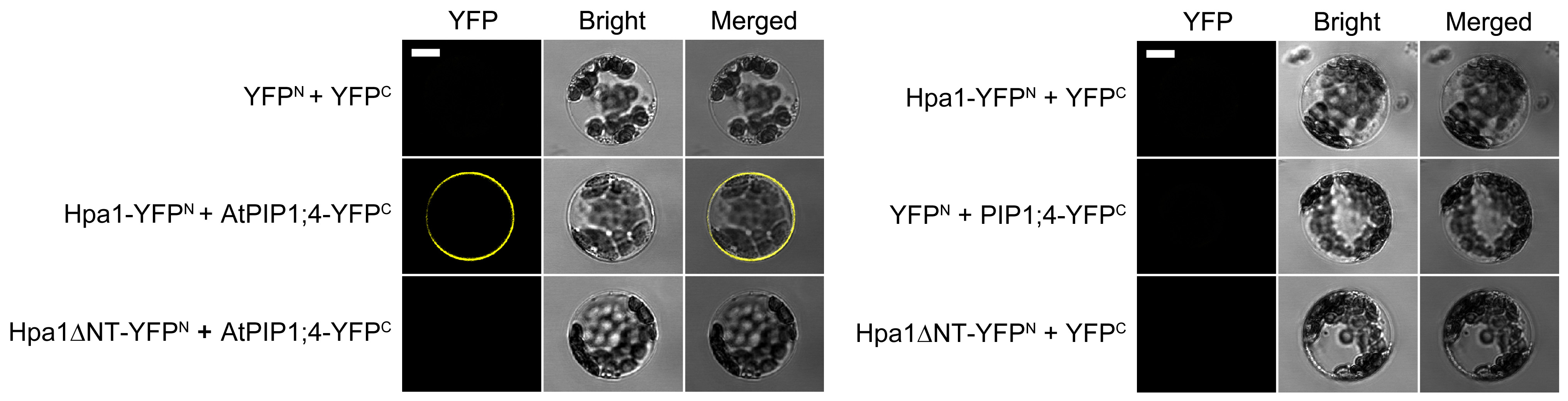
Supplementary Figure 4 | Protoplast BiFC assays of AtPIP1;4 and Hpal compared to multiple control proteins.** Protoplasts were observed by confocal microscopy at 60 hours after transformation. Scale bar = 10 μm.

**
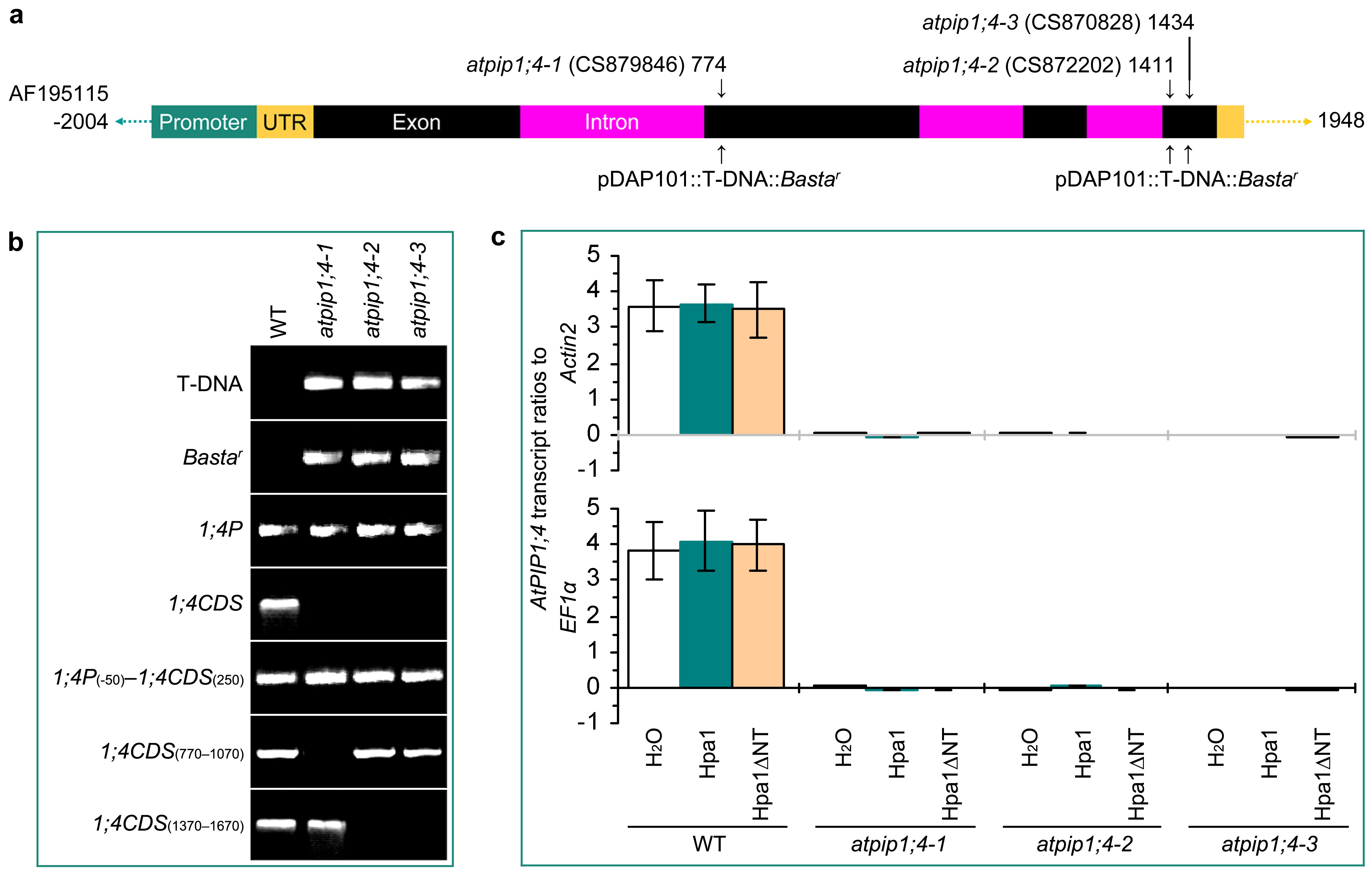
Supplementary Figure 5 | Insertional mutations of *AtPIP1;4* nullifies its expression. (a)** A map showing the pDAP101 vector that contains the selective marker gene *Basta^r^* (bottom labels) and location of T-DNA insertion in *atpip1;4* mutants. Seed stock numbers are in parentheses. Colored bars indicate the gene DNA components, including untranslated region (UTR). **(b)** Identification PCR of T-DNA-indexed *AtPIP1;4* mutations. The genomic DNA was analyzed by using primers specific to gene sequences indicated on left, including the T-DNA and *Basta^r^* inserts, as well as *AtPIP1;4* promoter (*1;4P*) and CDS (*1;4CDS*) of different spans in the nucleotide sequence. **(c)** Real-time RT-PCR analyses of RNAs from differently treated plants. The constitutively expressed *Actin2* and *EF1α* genes were used as references. Data shown are mean values ± SEM (standard error from mean) bars (*n* = 7 experimental repeats).

**
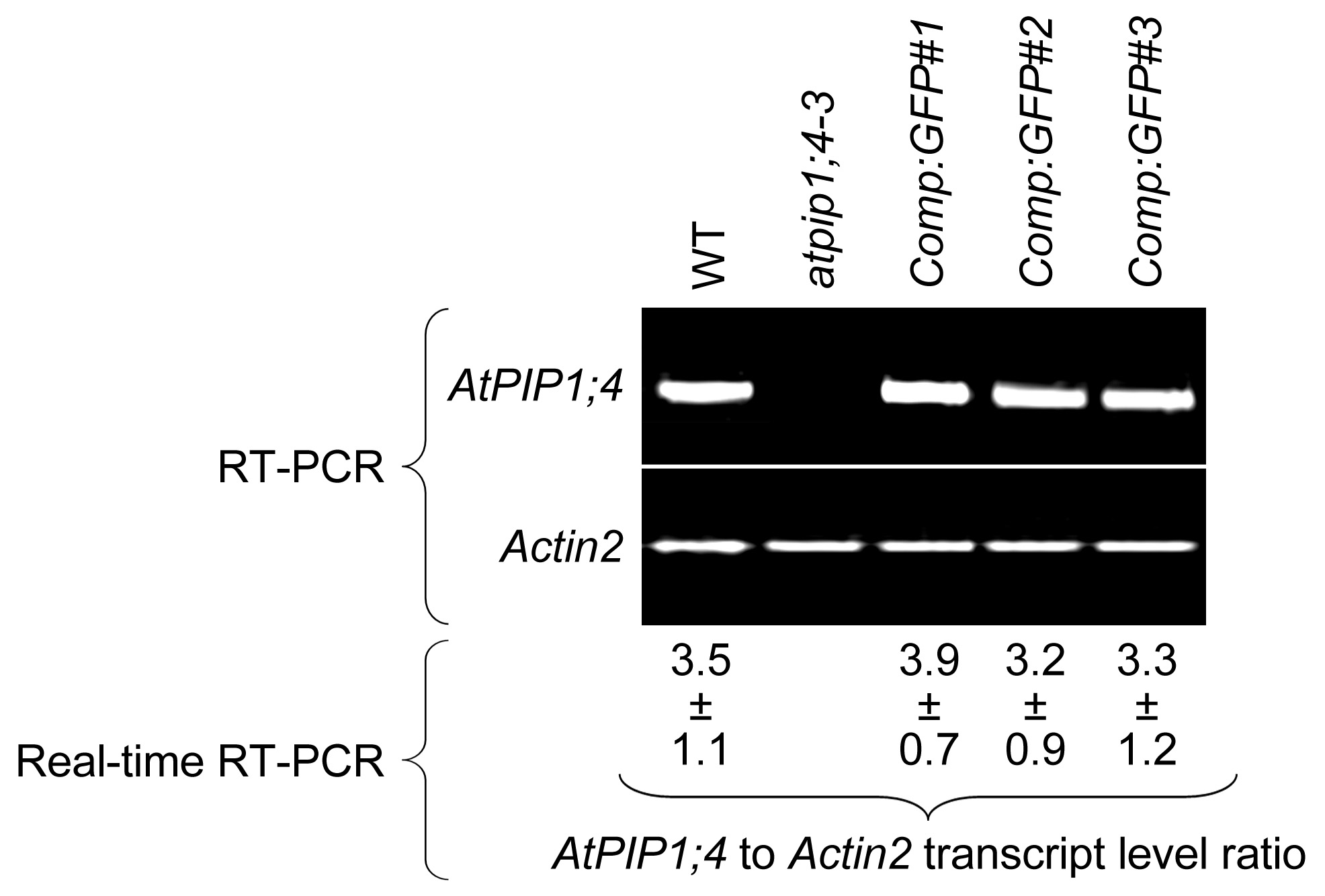
**

**Supplementary Figure 6 | Genetic complementation of *atpip1;4*.** The expression of *AtPIP1;4* was nullified in the *atpip1;4-3* mutant and reverted to approximations of the WT level in the genetically complementation (*Comp:GFP*) lines. The *AtPIP1;4* to *Actin2* transcript ratio was given as mean value ± SEM (*n* = 6 experimental repeats).

**
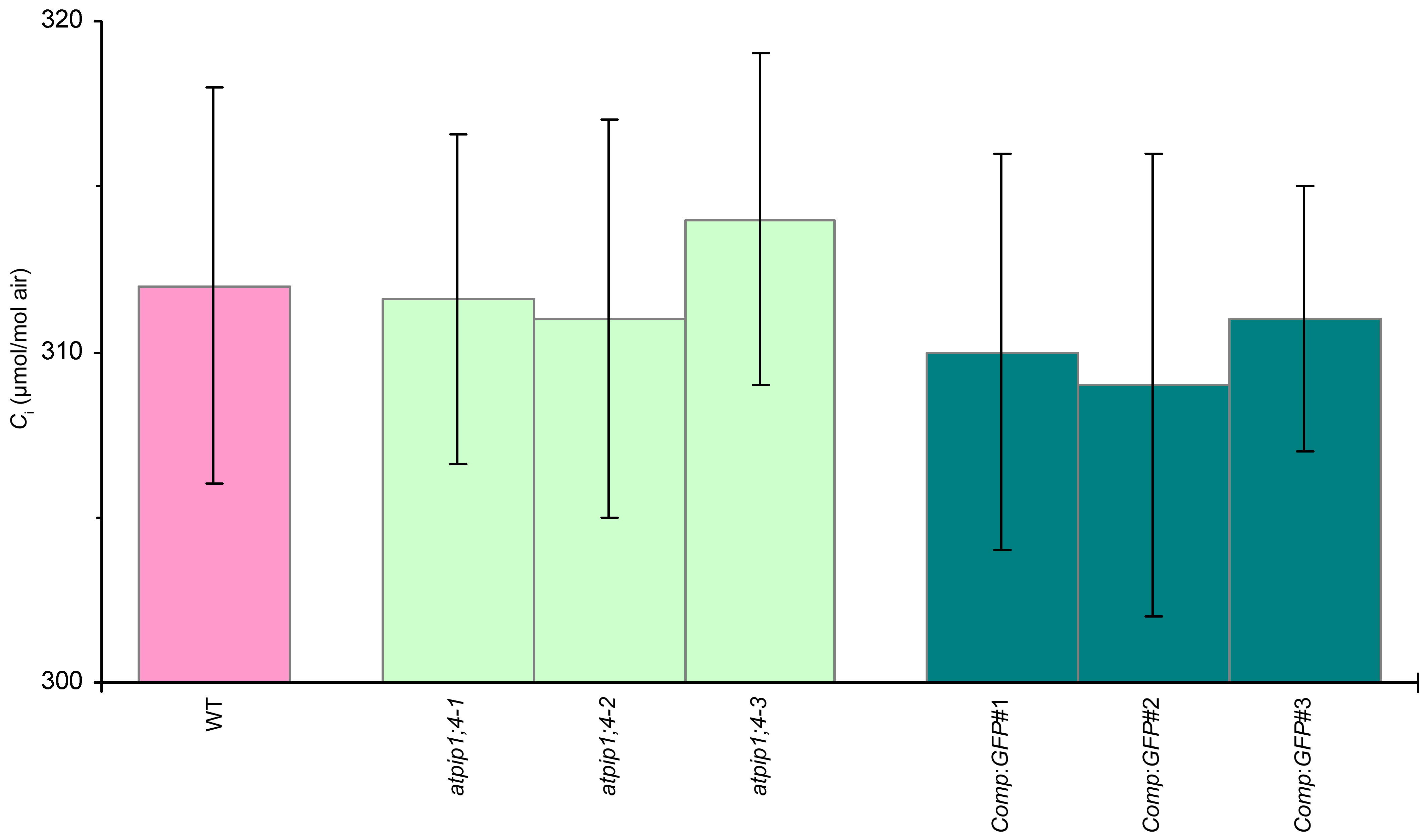
Supplementary Figure 7 | *g*_s_** **in WT, *atpip1;4*, and complemented plants.**Data shown are mean values ± SEMs (*n* = 18 leaves).

**
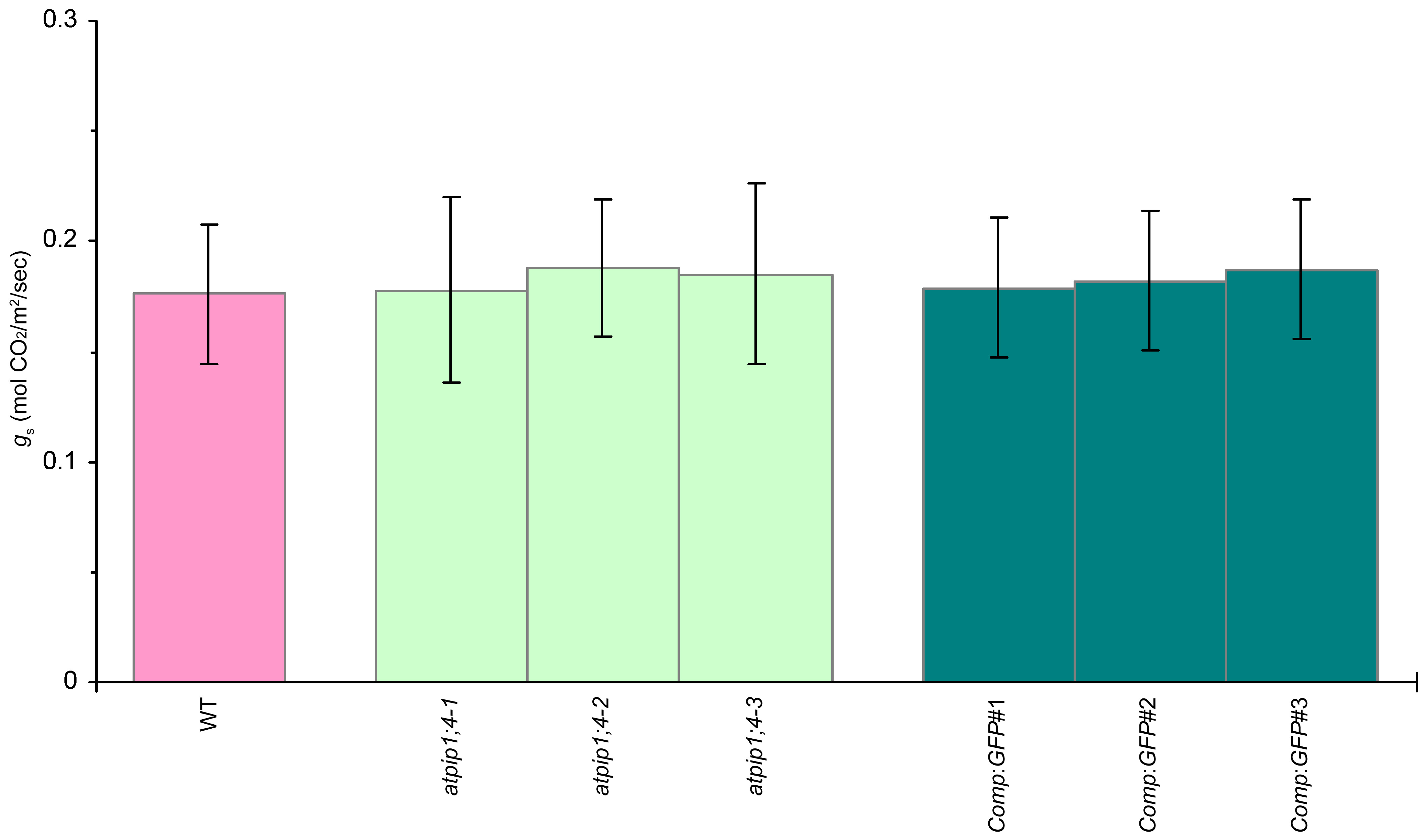
Supplementary Figure 8 | *C*_i_ in WT, *atpip1;4*, and complemented plants.** Data shown are mean values ± SEMs (*n* = 18 leaves).

**
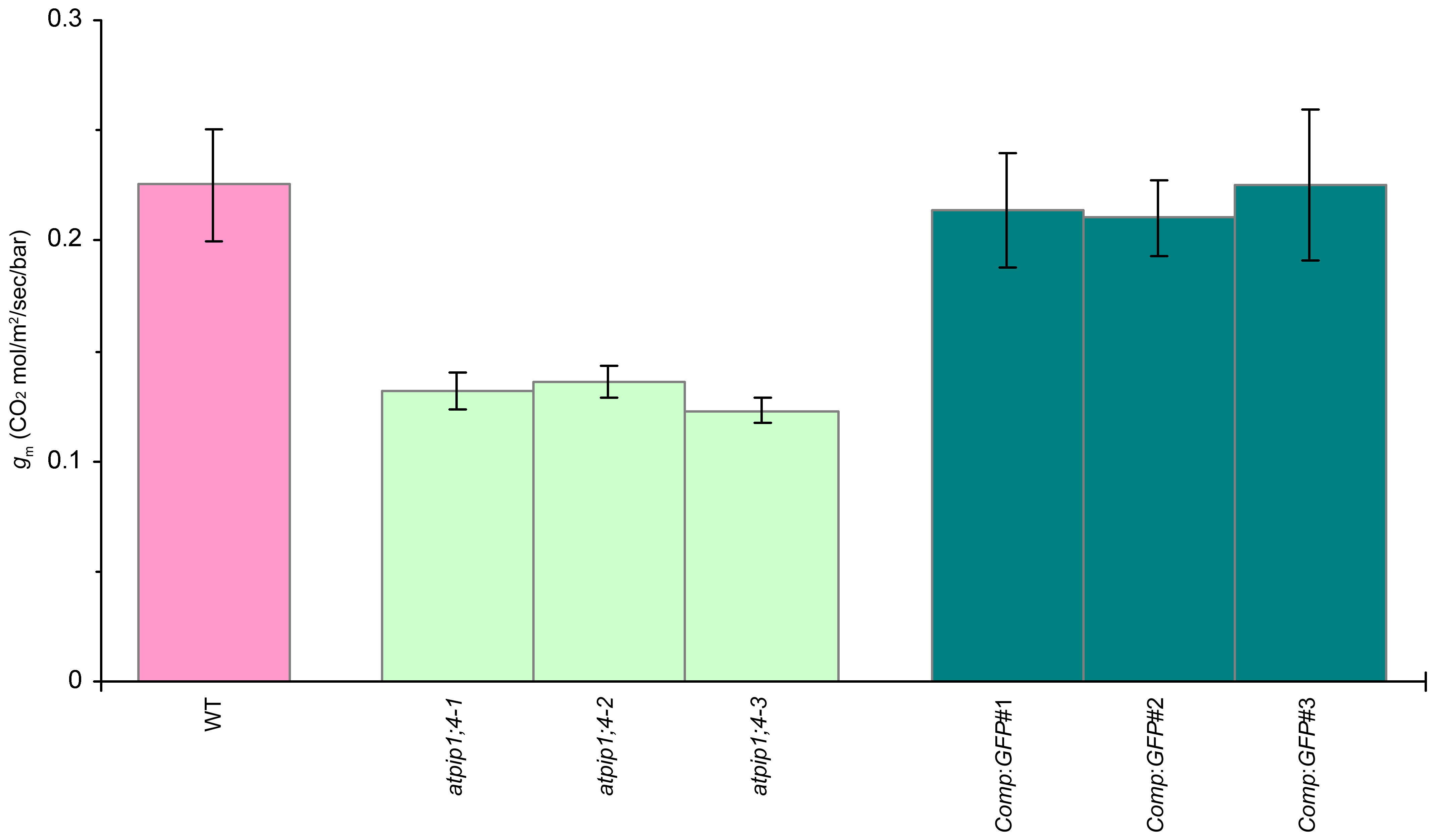
Supplementary Figure 9 | Arabidopsis *g_m_* estimates** **based on *A*_N_-*C*_i_ curve.** Data shown are means ± SEMs (*n* = 18 leaves).

**
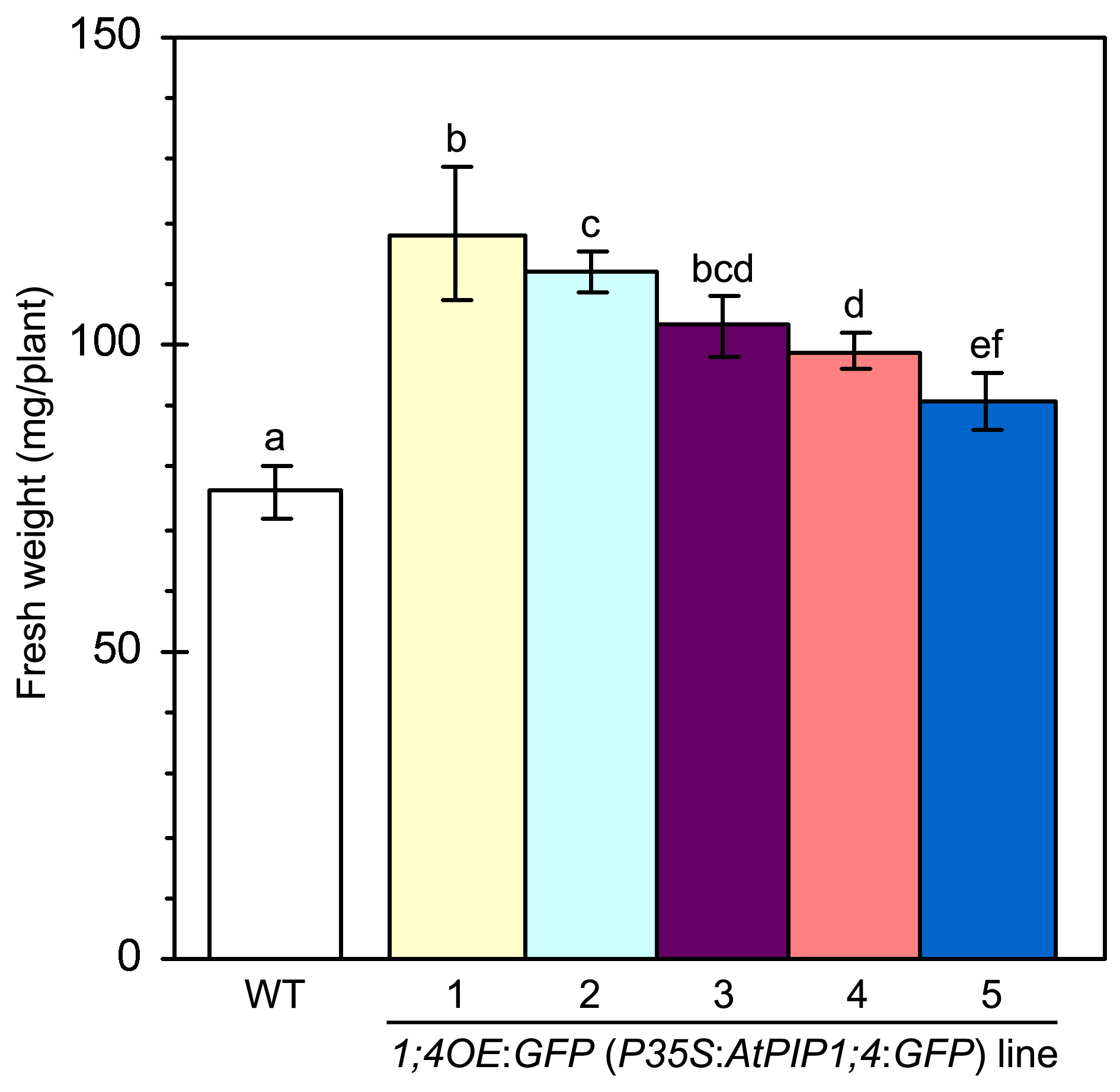
**

**Supplementary Figure 10 |** ***AtPIP1;4* overexpression enhances plant growth.** Fresh weight of 30-day-old plants was determined as means ± SEMs (*n* = 225 plants). Different letters on error bars indicate significant differences (*P* < 0.01).

**
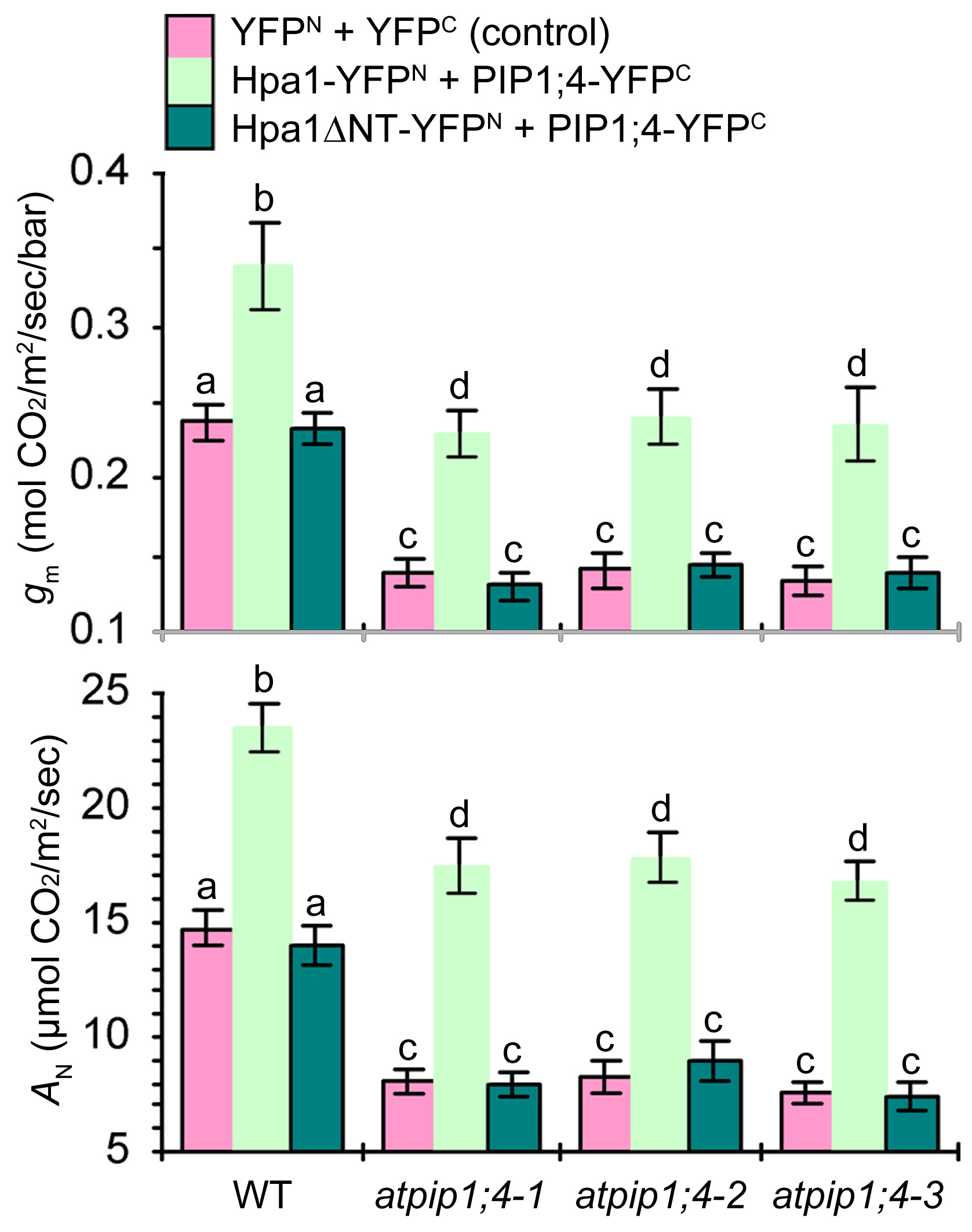
**

**Supplementary Figure 11 | *AtPIP1;4* mutation impairs *A*_N_, *g*_m_, and the promoting effect of Hpa1.** Data shown are means ± SEMs (*n* = 18 leaves). Different letters on error bars indicate significant differences (*P* < 0.01).

**Supplementary Table 1 | Photosynthetic parameters determined and used in *g*_m_ estimate based on gas exchange and ^13^C discrimination**

| Analysis | Parameters | Wild type | *atpip1;4-3* |
| --- | --- | --- | --- |
| Gas exchange | *A***_N_** (μmol/m^2^/s) | 14.376 ± 0.872 a | 7.542 ± 0.232 b |
|  | *C*_i_/*C*_a_ | 0.715 ± 0.002 a | 0.822 ± 0.005 b |
|  | Δ_i_ (‰) | 21.420 ± 0.056 a | 23.953 ± 0.112 b |
| Carbon isotope  discrimination | δ^13^C in leaf dry matter (‰) | -25.240 ± 0.112 a | -26.782 ± 0.182 b |
| *g*_m_ (mol/m^2^/s/bar) | | 0.220 ± 0.003 a | 0.121 ± 0.003 b |

Gas exchange in top 3–5 expanded leaves of 35-day-old plants was measured and using the same leaves, *g*_m_ was quantified by the isotope discrimination in recently synthesized carbohydrates. Values are means ± SEMs (*n* = 3 experimental repeats; 150 plants per repeat). Different letters on error bars indicate significant differences by analysis of variance method along with least significant difference test (*P* < 0.05).

**Supplementary Table 2 |** Arabidopsis cell pressure probe measurements

| Variable | Water | | Hpa1 | |
| --- | --- | --- | --- | --- |
| **Root cell** | Wild type | *atpip1;4-3* | Wild type | *atpip1;4-3* |
| Turgor pressure, TP (MPa) | 0.35 ± 0.03 a | 0.34 ± 0.02 a | 0.36 ± 0.19 a | 0.35 ± 0.16 a |
| Cell volume (m^3^) | 2.57E-14 | 2.72E-14 | 2.60E-14 | 2.70E-14 |
| Cell surface area (m^2^) | 5.76E-09 | 5.92E-09 | 5.80E-09 | 5.90E-09 |
| ε (MPa) | 2.4 ± 0.3 a | 2.2 ± 0.2 a | 2.3 ± 0.2 a | 2.3 ± 0.2 a |
| T_1/2_ (s) | 1.8 ± 0.3 a | 2.2 ± 0.6 a | 1.2 ± 0.2 b | 2.1 ± 0.5 a |
| Lp_rc_ (ms^-1^ MPa^-1^ × 10^-6^) | 0.72 ± 0.10 b | 0.62 ± 0.07 b | 1.08 ± 0.11 a | 0.67 ± 0.05 b |
| **Leaf cell** | Wild type | *atpip1;4-3* | Wild type | *atpip1;4-3* |
| Turgor pressure, TP (MPa) | 0.41 ± 0.04 a | 0.40 ± 0.03 a | 0.41 ± 0.05 a | 0.40 ± 0.05 a |
| Cell volume (m^3^) | 5.26E-13 | 5.13E-13 | 5.30E-13 | 5.10E-13 |
| Cell surface area (m^2^) | 4.61E-08 | 4.52E-08 | 4.60E-08 | 4.50E-08 |
| ε (MPa) | 4.2 ± 0.3 a | 4.1 ± 0.4 a | 4.1 ± 0.4 a | 4.2 ± 0.2 a |
| T_1/2_ (s) | 1.5 ± 0.2 b | 1.8 ± 0.2 a | 1.1 ± 0.1 c | 1.8 ± 0.3 a |
| Lp_lc_ (ms^-1^ MPa^-1^ × 10^-6^) | 1.22 ± 0.09 b | 1.04 ± 0.05 c | 1.67 ± 0.19 a | 1.09 ± 0.09 bc |

Data are means ± SD (*n* = 15 to 20 cells). Different letters indicate significant differences (*P* < 0.05).

**Supplementary Table 3.** Information on genes tested and primers used in this study

| Gene (code or source) | Primers / product size (bp) / subjects |
| --- | --- |
| *hpa1* (AY139029) | 5’-CG**GGATCC**ATGAATTCTTTGAACACACAATT-3’ (*Bam*H I), 5’-**GTCGAC**CTGCATCGATGCGCTGTCGC-3’ (*Sal* I)/ 420 / cloning by PCR |
|  | 5’- AA**GGCCATTACGGCC**ATGAATTCTTTGAACACACAA-3’, (*Sfi* I), 5’-TT**GGCCGAGGCGGCC**TTACTGCATCGATGCGCTGTCGC-3’ (*Sfi* I) / 420 / Y2H |
|  | Upstream homologous arm 5’-CG**GGATCC**CGCTGCTGCAAATAGAATACG-3’ (*Bam*H I), 5’-GTAGGGGCGACCAACAGTTCTCGTGACGATTCCTCTCTGATT-3’ / 537  Downstream homologous arm 5’-AATCAGAGAGGAATCGTCACGAGAACTGTTGGTCGCCCCTAC-3’, 5’-GC**TCTAGA**TCACTCGCACATGCTGGTTCT-3’ (*Xba* I) / 536 / deletion |
|  | 5’-AA**CTGCAG**TACCCAGGACCAACGAAACACTT-3’ (*Pst* I), 5’-GG**GGTACC**TTActtatcgtcgtcatccttgtaatc(flag)CTGCATCGATGCGCTGTCGCT-3’ (*Kpn* I) / 420 / secretion |
|  | 5’-GG**GGTACC**ATGAATTCTTTGAACACACAA-3’ (*Kpn* I), 5’-CG**GGATCC**CTGCATCGATGCGCTGTCGC-3’ (*Bam*H I) / 420 / BiFC |
| *hpa1∆159* | 5’-CG**GGATCC**ATGTCGAGCAAAAATGCTGAGG-3’ (*Bam*H I), 5’-**GTCGAC**CTGCATCGATGCGCTGTCGC-3’ (*Sal* I)/ 261 / production of the Hpa1∆NT protein |
|  | 5’-AA**GGCCATTACGGCC**ATGTCGAGCAAAAATGCTGAGG-3’ (*Sfi* I), 5’-TT**GGCCGAGGCGGCC**TTACTGCATCGATGCGCTGTCGC-3’ (*Sfi* I) / 261 / Y2H |
|  | 5’-GG**GGTACC**ATGTCGAGCAAAAATGCTGAG (*Kpn* I), 5’-CG**GGATCC**CTGCATCGATGCGCTGTCGC-3’ (*Bam*H I) / 261 / BiFC |
| Arabidopsis *Actin2* (X15864) | 5’-GTCTCCTGCAAGTCGGGGA-3’, 5’-CTAGGGAAAACAGCACGG-3’ / 207 / real-time RT-PCR |
| *AtPIP1;4* (AT4G00430) | 5’-GG**GGTACC**ATGGAAGGCAAAGAAGAA-3’ (*Kpn* I), 5’-CG**GGATCC**ACTCTTGCTC TTGAAAGGA-3’ (*Bam*H I)/ 864 / CDS cloning by RT-PCR and overexpression construction |
|  | 5’-CATGCTCACCCTCGATTGTTC-3’, 5’-CTCCCATTACAGTCAAAAC-3’ / 205 / real-time RT-PCR |
| *AtPIP1;4* (AF195115.1) | 5’-GC**GAATTC**CAAATTTTTACTCATGGTTTTTGCAC-3’ (*Eco*R I), 5’-**GTCGAC**TGTGGTCGAGACTTCATTTATA-3’ (*Sac* I) / 2004 / promoter cloning by PCR |
| *RFP* (AY640628) | 5’-GC**TCTAGA**ATGGCCTCCTCCGAGAACGTCATC-3’ (*Xba* I), 5’-CG**GGTACC**CTACAGGAACAGGTGGTGGCGGC-3’ (*Kpn* I) / 694 / recombination |
| T-DNA (pROK2, pCSA110 or pDAP101) | 5’-CTCTGTCATCGTTACAATCAACA-3’, 5’-CCAATACATTACACTAGCATCTG-3’ / 373 / verification of *AtPIP1;4* sequence-indexed T-DNA insertion |
| *Kan^r^* (as above) | 5’-CTGGGCACAACAGACAATC-3’, 5’-CAGAAGAACTCGTCAAGAAG-3’ / 726 / the same as above |
| *Basta^r^* (as above) | 5’-CTGCACCATCGTCAACCAC-3’, 5’-AAGTCCAGCTGCCAGAAAC-3’ / 441 / the same as above |
| *AtPIP1;4P* (AF195115.1) | 5’-GCCAAATTTTTACTCATGGTTTTTGCAC-3’, 5’-TGTGGTCGAGACTTCATTTATA-3’ / 2004 / the same as above |
| *AtPIP1;4CDS* (AT4G00430) | 5’-ATGGAAGGCAAAGAAGAAGATG-3’, 5’-TCAGCAAACCGGAACTAAGAGA-3’ / 660 / verification of *AtPIP1;4* sequence-indexed T-DNA insertion |
| *AtPIP1;4P*_(-50)_**–***CDS*_(250)_ (AT4G00430) | 5’-GGAGAGAGAAGAACAAAAG-3’, 5’-TCCAGCAGTACAGTAGACA-3’ / 301 / the same as above |
| *AtPIP1;4CDS*_(770-1070)_ (AT4G00430) | 5’-AAAGGTTTCCAGCCAACGCC-3’, 5’-CTGCAAATCCGATTGGGAGC-3’ / 301 / the same as above |
| *AtPIP1;4CDS*_(1370-1670)_ (AT4G00430) | 5’-CACTCTAATGAAAAGGACAACTCG-3’, 5’-GCATAGGAAGAAAAAAGAAACACC-3’ / 301 / the same as above |
| *YFP* (AY189981) | 5’-GC**TCTAGA**ATGGTGAGCAAGGGCGAGGAG-3’ (*Xba* I), 5’-CG**CTGCAG**TTAAGATCTGTACAGCTCGTC-3’ (*Pst* I)/ 723 /, 5’-CG**GGTACC**CTACAGGAACAGGTGGTGGCGGC-3’ (*Kpn* I) / 694 / recombination |
